# Abscopal effect of focused ultrasound combined immunotherapy in animal solid tumor model: a systematic reviews and meta-analysis

**DOI:** 10.3389/fimmu.2024.1474343

**Published:** 2024-12-13

**Authors:** Chao Hu, Hui Li, Tao Deng, Zheng Liu, Li Yang, Li Peng, Ming Yan Jiang, Wen Zhi Chen

**Affiliations:** ^1^ State Key Laboratory of Ultrasound in Medicine and Engineering, Chongqing Medical University, Chongqing, China; ^2^ Department of Pulmonary and Critical Medicine, Xiangtan Central Hospital, Xiangtan, China; ^3^ Department of Pharmaceutical, Xiangtan Central Hospital, Xiangtan, China; ^4^ Department of Oncology, Xiangtan Central Hospital, Xiangtan, China

**Keywords:** abscopal effect, focused ultrasound, immunotherapy, meta-analysis, systematic review

## Abstract

**Introduction:**

The abscopal effect, a systemic anti-tumor response triggered by localized treatment, has gained attention but remains poorly understood. This study evaluates the efficacy and consistency of focused ultrasound (FUS) combined with immunotherapy in inducing the abscopal effect.

**Methods:**

A systematic review and meta-analysis were conducted on preclinical studies using solid tumor models. Data on tumor response, immune modulation, and survival outcomes were analyzed to assess the combination therapy's effectiveness.

**Results:**

FUS combined with immunotherapy enhanced anti-tumor responses at local and distant sites, with evidence of immune activation and increased abscopal effect rates. However, heterogeneity across tumor models and protocols was observed.

**Discussion:**

The findings provide a theoretical basis for FUS-immunotherapy combinations in cancer treatment, while emphasizing the need for standardized protocols and further research to elucidate underlying mechanisms.

**Systematic review registration:**

https://www.crd.york.ac.uk/prospero/, identifier CRD42023460710.

## Graphical Abstract

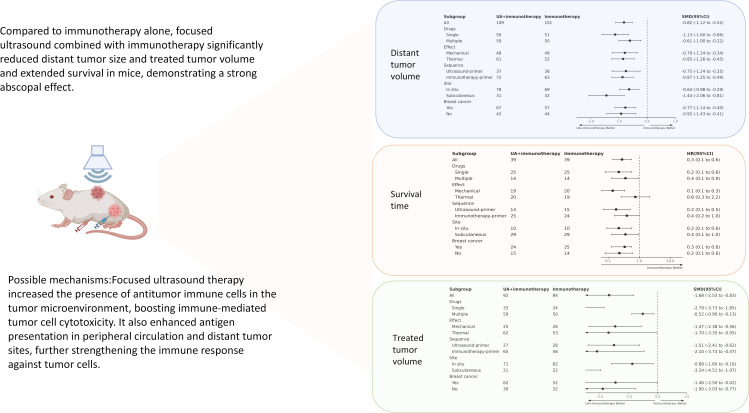

## 1 Introduction

First identified in radiotherapy, the abscopal effect describes the regression or reduction of tumors outside the irradiated field (1). Owing to its unpredictable nature, this phenomenon has mostly been observed in case reports and early-phase single-arm clinical trials (2). The emergence of immunotherapy has enabled the combination of local and systemic treatments, thus increasing the incidence of the abscopal effect (3) and generating enthusiasm for using combination therapies in both localized and metastatic cancers (4, 5). However, the exact mechanisms underlying the abscopal effect remain unclear because of the variety of local treatment methods.

Different therapies may trigger distinct processes, such as the production of tumor cell fragments, release of tumor antigens, activation of the immune system, or alteration of the tumor microenvironment. For example, radiotherapy can damage tumor cell DNA, leading to the release of tumor antigens and cytokines, which subsequently activate dendritic cells and T cells, resulting in a systemic anti-tumor response (6–8). As a precise and non-invasive local treatment modality, focused ultrasound (FUS) can effectively destroy tumor cells while also activating an immune response (9, 10); thus, it has been widely applied in treating of solid tumors. However, FUS alone is often insufficient to elicit a robust anti-tumor immune response capable of inducing a significant abscopal effect (11, 12). Preclinical models by Han et al. have demonstrated that FUS treatment has minimal impact on the immune microenvironment of distant tumors (13), with no significant inhibition of tumor growth observed post-treatment. This has led to an interest in combination therapies.

FUS could induce the release of tumor-associated antigens, activating anti-tumor immunity. Immunotherapy could enhances the recognition and processing of these antigens, prompting immune cells to target and destroy tumor cells (14–16). Together, these treatments work synergistically to reduce the likelihood of tumor recurrence and metastasis. However, studies investigating the abscopal effect of combined FUS and immunotherapy are relatively sparse, and the precise mechanisms and consistency of this effect remain largely unconfirmed, which hinders the clinical application of combined treatments. Therefore, this study aims to conduct a systematic review and meta-analysis of existing preclinical animal models of solid tumors to evaluate the efficacy and consistency of FUS combined with immunotherapy in achieving the abscopal effect. Our findings provide a more robust theoretical foundation and data support for future clinical research.

## 2 Methods

This systematic review adhered to the Preferred Reporting Items for Systematic Reviews and Meta-Analyses (PRISMA) guidelines and the Systematic Review Centre for Laboratory animal Experimentation (SYRCLE) tool for animal studies. The protocol has been registered with PROSPERO (registration no. CRD42023460710).

### 2.1 Search strategy

We conducted a comprehensive search of English-language database, namely Medline, Embase, and Web of Science, and Chinese-language databases, namely SinoMed, CNKI, and Wanfang from January 1, 2001, to October 14, 2024. The search was limited to English/Chinese-language publications. The strategy comprised three components: focused ultrasound, solid tumors, and immunotherapy. Results were restricted to animal studies. The complete search strategy is summarized in **Supplementary Table 1**.

### 2.2 Inclusion and exclusion criteria

Studies were deemed eligible based on the following criteria: (1) the subjects were animal models of solid tumors, (2) interventions included both immunotherapy and FUS, (3) a control group with immunotherapy alone was present, (4) outcomes included tumor volume or survival time, and (5) the study was published in English or Chinese. The exclusion criteria were as follows: (1) non-original or incomplete research articles; (2) reviews, retrospective studies, and protocols; (3) studies lacking at least two solid tumors to evaluate the abscopal effect; (4) studies with undefined sample sizes; and (5) ultrasound modalities such as sonodynamic therapy or drug delivery.

### 2.3 Data extraction

Two reviewers conducted independent data extraction from the chosen studies. Any inconsistencies were addressed through consultation with a third reviewer. Baseline characteristics included the following: (1) publication specifics (author and year); (2) interventions (FUS device, treatment effects and sessions, type, dose, route, and timing of immunotherapy); (3) tumor cell type, site, and method of modeling; (4) animal characteristics (species, strain, age, and weight); and (5) outcomes. All outcome data were continuous. We extracted sample size (N), median values, and 95% confidence intervals for each group. When results were presented in graphical form, quantification was performed using the WebPlot Digitizer software.

### 2.4 Quality assessment

Two reviewers independently assessed the internal validity of included publications using the SYRCLE risk of bias tool for animal experiments. The six-domain checklist covered the following:(1) selection bias (sequence generation, baseline characteristics, and allocation concealment); (2) performance bias (randomization and blinding); (3) detection bias (random outcome assessment and blinding of outcome assessors); (4) attrition bias (incomplete outcome data); (5) reporting bias (selective outcome reporting); and (6) other biases (model assessment, temperature control, pharmaceutical manufacturing, conflicts of interest). Disagreements were resolved through discussion with a third reviewer.

### 2.5 Statistical analysis

Data analysis and visualization were performed using STATA (version 15.1, Stata Corp LLC, College Station, TX, USA), Review Manager (version 5.0, The Cochrane Collaboration, Oxford, UK), and MedCalc software (version 22.009, MedCalc Software Ltd, Ostend, Belgium). Standardized mean differences (SMD) were used as the effect measure for tumor volume outcomes, and hazard ratios (HR) were used for survival time. If studies included multiple independent groups (e.g., different animal models or time points), they were treated as separate experiments. Heterogeneity was assessed using the *I^2^* and *Q* tests; a fixed-effects model was used if *I^2^* < 50%, whereas a random-effects model was used if *I^2^* ≥ 50%. Subgroup analyses explored the sources of heterogeneity and diversity, considering factors such as combination immunotherapy drugs, types of FUS effects, treatment sequence, and tumor models. Publication bias was assessed by visual inspection of funnel plots and the Egger test.

## 3 Results

### 3.1 Study selection

**Figure 1:**
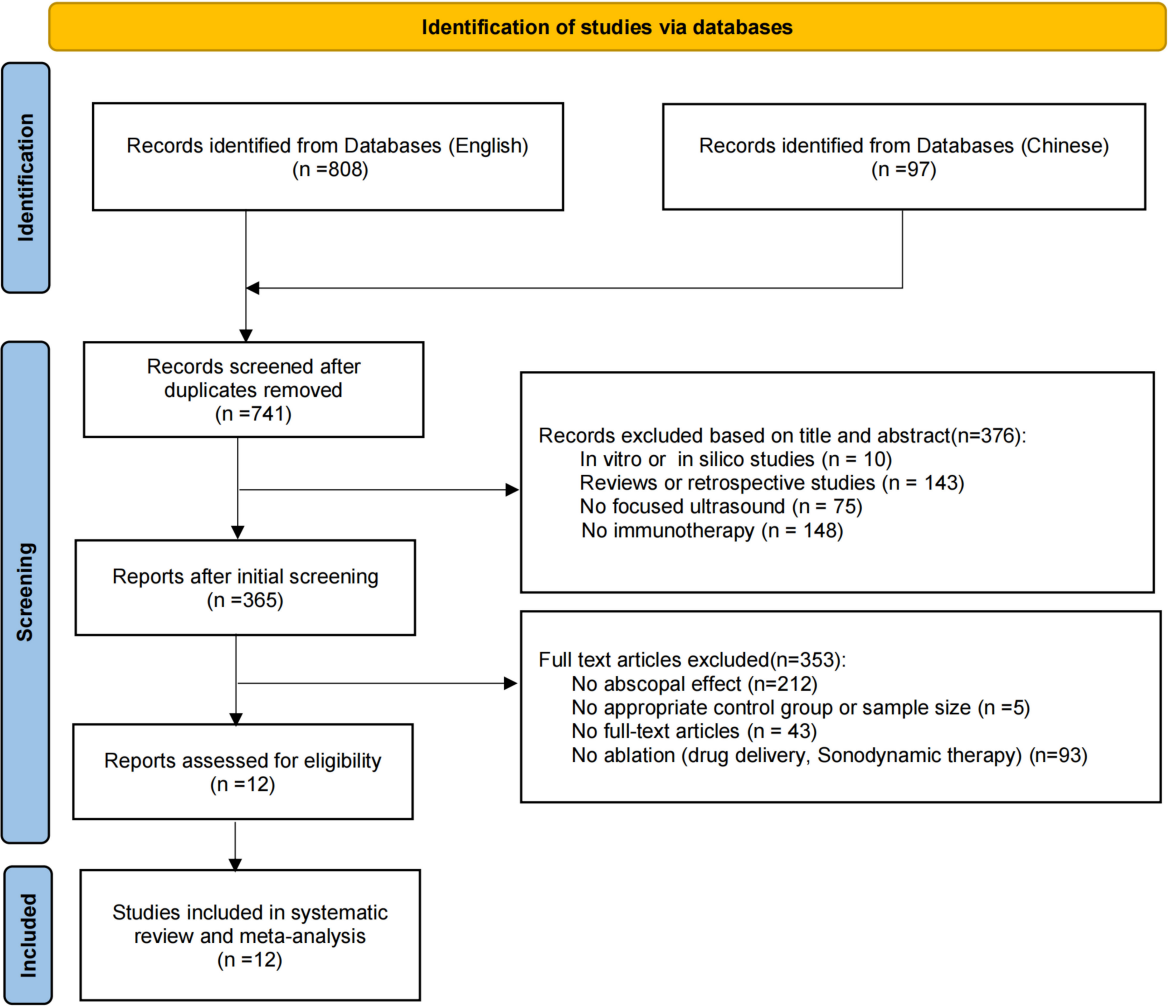
Flowchart of the study selection process. A systematic search was conducted using English databases (Medline, Embase, Web of Science) and Chinese databases (SinoMed, CNKI, and Wanfang), covering the period from January 1, 2001, to October 14, 2024.

A comprehensive search yielded 905 studies, of which 164 were excluded as duplicates. Based on the exclusion criteria, 376 studies were removed after screening titles and abstracts. Full-text evaluation led to the exclusion of 353 studies, resulting in 12 eligible publications. The study selection process is depicted in **Figure 1**.

### 3.2 Study characteristics

A total of 12 articles (17–28) were included in the analysis. All studies were conducted between 2017 and 2024, and exclusively used mice. Of these, 7studies used C57BL/6 mice, 3 used BALB/c mice, and 2 used FVB/n mice. The immunotherapy agents employed included anti-PD-1/L1, anti-CTLA-4, CD40, and CpG agonists, among others, with varying administration frequencies depending on the treatment regimen. FUS utilized both thermal and mechanical effects. Considering the primary sites of breast cancer and melanoma, two studies used breast as the transplantation site and two used skin for melanoma. Tumor types included breast cancer (n=5), followed by melanoma (n=4), colorectal cancer (n=2), and pancreatic(n=1) and liver cancers (n=1). Key outcomes measured were abscopal tumor volume, overall survival time, and primary tumor volume. Detailed characteristics are summarized in **Table 1**.

Table 1Characteristics of all included studies.

**Table:** 

Author	Year	Strain/Species	Experimental group	Control group	Gender	Age	Animal number:exp/c	Type of animal model	Tumor location	Immunotherapy
Michael Chavez	2018	FVB/n mice	UA+CPG+anti-PD-1	CPG+anti-PD-1	F	4-5w	45/35	NDL,breast cancer	the fourth and ninth inguinal mammary fat pads	CPG;anti-PD-1;
Jiawei Tang	2023	C57BL/6mice	UA+anti-PD-1	anti-PD-1	F	6-8w	5/5	MC38,colon cancer	bilateral flank	anti-PD-1
Matthew T. Silvestrini	2017	FVB/n mice	UA+CPG+anti-PD-1	CPG+anti-PD-1	F	6-10w	5/5	NDL,breast cancer	the fourth and ninth inguinal mammary fat pads	CPG;anti-PD-1;
James Wang	2022	C57BL/6J mice	UA+CD40+anti-PD-1+anti-CTLA-4	CD40+anti-PD-1+anti-CTLA-4	F	4w	5/4	MT4,pancreatic cancer	bilaterally hind flank	CD40;anti-PD-1;anti-CTLA-4;
Mohit Pratap Singh	2019	C57/6J mice	UA+CD40	CD40	NA	NA	6/6	B16F10,melanoma	bilateral flank	anti-CD40;
Shi Bin Qu	2020	C57/6J mice	UA+anti-CTLA-4	anti-CTLA-4	NA	6-8w	4/4	B16F10-GP33,melanoma	bilateral flank	anti-CTLA-4
Shinya Abe	2022	BALB/c mice	UA+anti-PD-L1	anti-PD-L1	NA	5-8w	10/10	MM3MG-HER2,breast cancer	left leg and right flank	anti-PD-L1;
Mohit Pratap Singh A	2021	C57BL/6 mice	UA+CD40	CD40	M	6-8w	5/5	B16F10,melanoma	bilateral flank	CD40;
Shi Bin Qu A	2020	C57/6J mice	UA+anti-CD40	anti-CD40	NA	6-8w	4/4	Hepa1-6,hepatocellular carcinoma	bilateral flank	anti-CTLA-4;
Shinya Abe A	2022	*hHER3+*BALB/c mice	UA+anti-PD-L1	anti-PD-L1	NA	5-8w	7/7	JC-HER3,breast cancer	left leg and right flank	anti-PD-L1;
Mohit Pratap Singh B	2021	C57BL/6 mice	UA+anti-CTLA-4+anti-PD-L1	anti-CTLA-4+anti-PD-L1	M	6-8w	4/5	B16F10,melanoma	bilateral flank	anti-PD-L1;anti-CTLA-4;
Mohit Pratap Singh C	2021	C57BL/6 mice	UA+CD40+anti-PD-L1+anti-CTLA-4	CD40+anti-PD-L1+anti-CTLA-4	M	6-8w	5/5	B16F10,melanoma	bilateral flank	CD40; anti-PD-L1; anti-CTLA-4;
Ashley L. Pepple	2023	C57BL/6 mice	UA+anti-CTLA-4	anti-CTLA-4	M	6-9w	4/4	B16F10,melanoma	bilateral flank	anti-CTLA-4;
Ting-Chuan Li	2020	C57BL/6 mice	UA+OK-432	OK-432	F	9w	5/6	CT27,colorectal adenocarcinoma	bilateral flank	Picibanil (OK-432)
Xinping Kuai	2022	BALB/c mice	MBP+HIFU	MBP	F	4w	5/5	4T1,breast cancer	Right hip and lung metastasis	MBP
Jingnan Li	2024	BALB/c mice	AFNMOFS+HIFU	AFNMOFS	F	NA	6/6,10/10	4T1,breast cancer	bilateral flank	AFNMOFS

**Table:** 

Author	Dosage	Timing of immunotherapy	Total dosage	Focused ultrasound	Equipment	Parameter	Timing of focused ultrasound	Combination therapy	Outcome measures
Michael Chavez	100ug/dose/tumor(i.m.);200ug/dose/mice(i.p.);	d21/24/28/31;d21/28/34;	800ug; 600ug;	thermal	Bruker BioSpec 7T small animal MR system	3MHZ	d31	immunotherapy-primer	tumor volume
Jiawei Tang	200ug/dose/mice(i.p.)	d4/7/10/13	800ug	mechanical	Ultrasound Needle	25.72KHZ	d9	immunotherapy-primer	survival rate, tumor volume
Matthew T. Silvestrini	100ug/dose/tumor(i.m.);200ug/dose/mice(i.p.);	d21/24/28/31/38/45;d21/28/35;	1200ug; 600ug;	thermal	Bruker BioSpec 7T small animal MR system	3MHZ	d31/38/45	immunotherapy-primer	survival rate, tumor volume
James Wang	100ug/dose/tumor(i.m.);200ug/dose/mice(i.p.);200ug/dose/mice(i.p.);	d11;d5/7/11;d5/7/11;	200ug; 600ug; 600ug;	thermal	Bruker BioSpec 7T small animal MR system	3MHZ	d11	immunotherapy-primer	survival rate
Mohit Pratap Singh	50ug/dose/tumor(i.m.);	d10/13/16/20	400ug	thermal	alpinion VIFU-2000	1.5MHZ	d10/13/16	Ultrasound-primer	tumor volume
Shi Bin Qu	200ug/dose/mice(i.p.);	d3/6/9/12	800ug;	mechanical	A field-programmable gate array development board	1MHZ	d8	immunotherapy-primer	tumor volume
Shinya Abe	200ug/dose/mice(i.p.);	d10/13/16	600ug;	mechanical	alpinion VIFU-2000	1.5MHZ	d7	Ultrasound-primer	tumor volume
Mohit Pratap Singh A	50ug/dose/tumor(i.m.);	once	100ug	mechanical	alpinion VIFU-2000	1.5MHZ	NA	Ultrasound-primer	survival rate, tumor volume
Shi Bin Qu A	200ug/dose/mice(i.p.);	d6/9/12	600ug;	mechanical	A field-programmable gate array development board	1MHZ	d10	immunotherapy-primer	tumor volume
Shinya Abe A	200ug/dose/mice(i.p.);	d8/11/15/18	800ug;	mechanical	alpinion VIFU-2000	1.5MHZ	d8	Ultrasound-primer	tumor volume
Mohit Pratap Singh B	200ug/dose/mice(i.p.);100ug/dose/mice(i.p.);	Three doses of ICl at 3 days interval;	NA;	mechanical	alpinion VIFU-2000	1.5MHZ	NA	Ultrasound-primer	survival rate, tumor volume
Mohit Pratap Singh C	50ug/dose/tumor(i.m.);200ug/dose/mice(i.p.);100ug/dose/mice(i.p.);	once; Three doses of ICl at 3 days interval;	100ug; NA;NA;	mechanical	alpinion VIFU-2000	1.5MHZ	NA	Ultrasound-primer	survival rate, tumor volume
Ashley L. Pepple	200ug/dose/mice(i.p.);	d6/9/12	600ug;	mechanical	alpinion VIFU-2000	1.5MHZ	d7	immunotherapy-primer	tumor volume
Ting-Chuan Li	0.25 Klinische einheit (KE)/dose/tumor(i.m.);	d0/3/6/9	2Klinische einheit	thermal	US machine (ITO Co., Tokyo, Japan)	1MHZ	d0/3/6/9	immunotherapy-primer	tumor volume
Xinping Kuai	10 mg/kg of Mn	Nine doses at 1 day interval	NA;	NA	NA	8.5w,20s	d7	immunotherapy-primer	tumor volume
Jingnan Li	an equal AQ4N dose of 10 mg/kg	D0/d5/d10	NA	thermal	JC200(Chongqing, China)	120w,1s	D1/d6/d11	immunotherapy-primer	survival rate, tumor volume

N/A, Not Applicable..

### 3.3 Quality assessment

**Figure 2:**
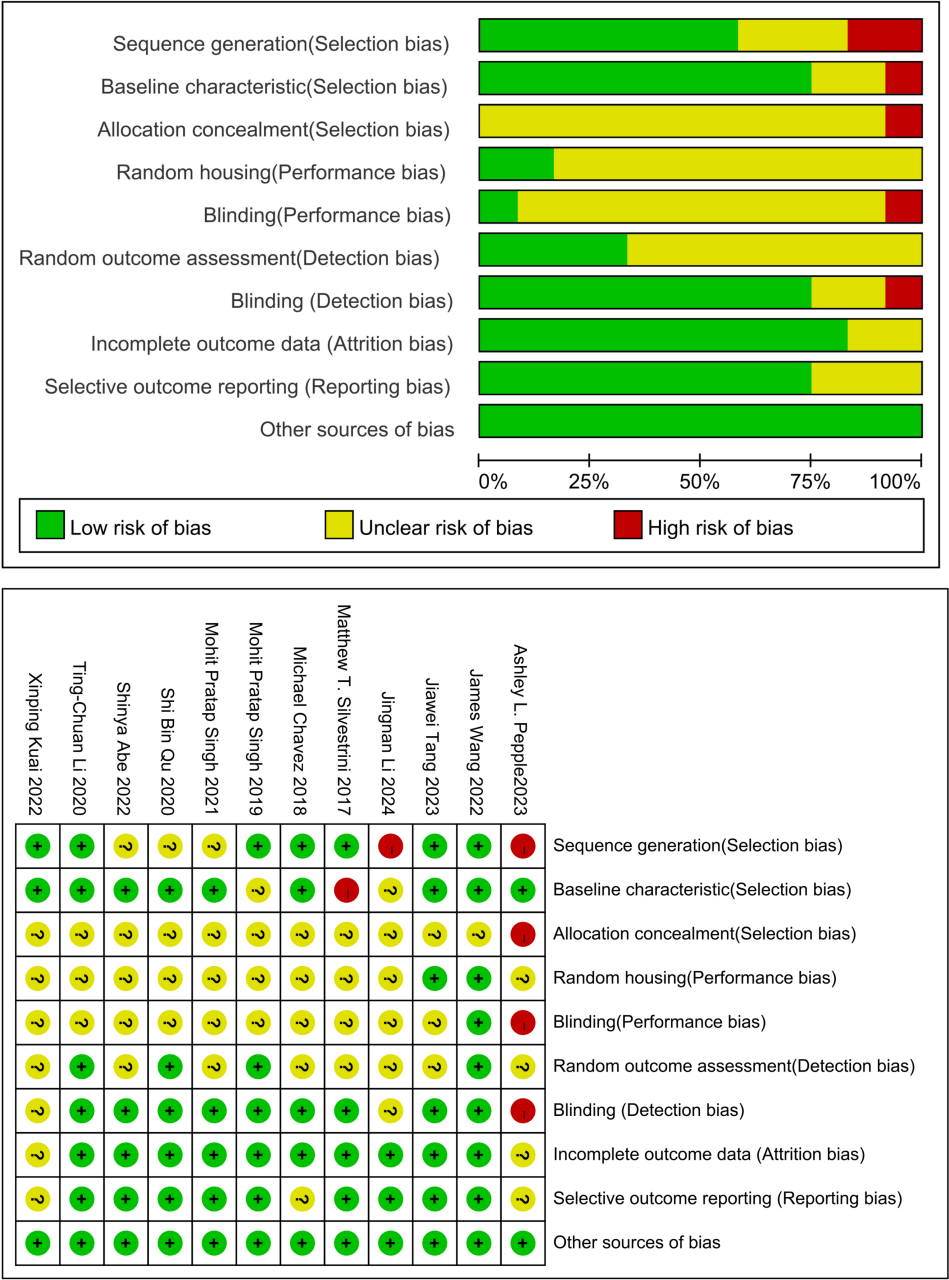
Study quality assessed using SYRCLE’s animal study bias tool. The quality assessment checklist comprises six components: (1) selection bias (sequence generation, baseline characteristics, and allocation concealment); (2) performance bias (randomization and blinding); (3) detection bias (randomized outcome assessment and blinding of outcome assessors); (4) attrition bias (incomplete outcome data); (5) reporting bias (selective outcome reporting); and (6) other biases (model evaluation, temperature control, pharmaceutical manufacturing institutions, conflicts of interest, etc.).

**Figure 2** presents the risk of bias assessment for the 12 included studies. All studies showed low risk in other bias categories. Most studies (11/12,91.67%) exhibited an unclear risk of bias in random sequence generation, and 83.33%(10/12) had a low risk concerning incomplete outcome data (17–28). However, the majority had unclear risks of bias related to allocation concealment, random housing, blinding, and random outcome assessment. None of the studies mentioned adequate allocation concealment. Although the overall quality of publications was suboptimal, no studies were excluded based on quality issues.

### 3.4 Main results

#### 3.4.1 Distant tumor volume

**Figure 3:**
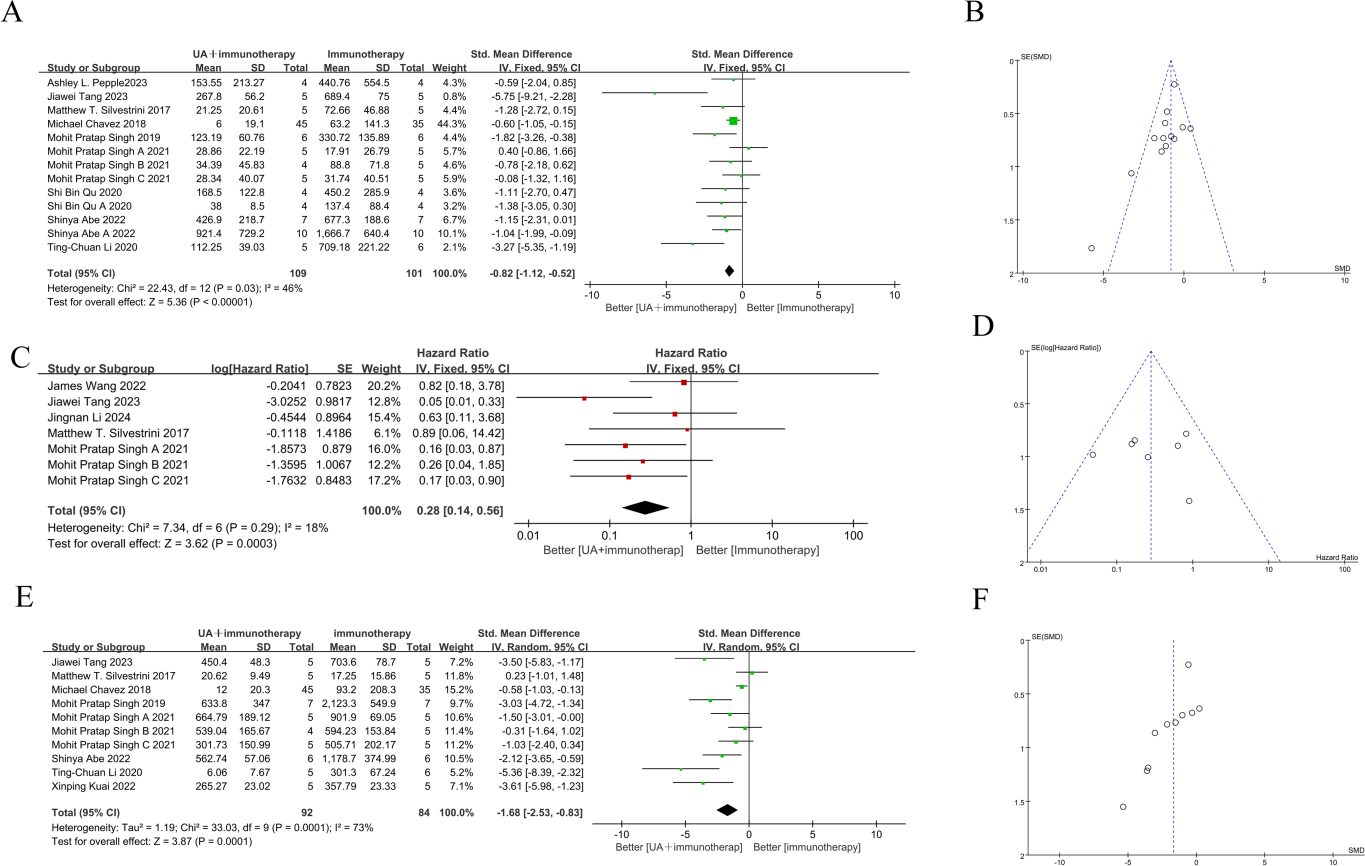
Forest plots and funnel plots of the main outcome. On the left side of the forest plot, the combined group (focused ultrasound therapy + immunotherapy) demonstrated greater benefit, while the right side indicated superior outcomes for the immunotherapy-only group. **(A)** Forest plots for distant tumor volume summarize the effect size of the standard mean difference, favoring the combined group, with the confidence intervals not crossing zero. **(B)** Funnel plots of distant tumor volume. **(C)** Forest plots for survival time summarize hazard ratios (HR), with values on the left side, indicating a benefit from the combined group, and confidence intervals not crossing one. **(D)** Funnel plots of survival time. **(E)** Forest plots for treated tumor volume summarize the effect size of the standard mean difference, favoring the combined group, with confidence intervals not crossing zero. **(F)** Funnel plots of treated tumor volume. The summary effect size is represented by ◆ (focused ultrasound therapy: UA).

The abscopal effect was evaluated based on the volume of distant tumors not treated with FUS. Of the 12 included studies, 10used abscopal tumor volume as an outcome measure, encompassing 14 independent experiments. The results indicated that the mean abscopal tumor volume in the focused ultrasound combined with immunotherapy group was lower than that in the immunotherapy alone group (SMD = -0.82 [95% CI −1.12, −0.52]; p < 0.00001, heterogeneity: *X²* = 22.43, *I²* = 46%, **Figure 3A**).

#### 3.4.2 Overall survival time

Five of the 12studies (7 independent experiments) reported overall survival time as an outcome measure. Meta-analysis of these experiments showed that the combination of FUS and immunotherapy significantly improved overall survival time in mice (HR = 0.28[95% CI 0.14-0.56]; *p*=0.0003, heterogeneity: *X²* = 7.34, *I²* = 18%, **Figure 3C**).

#### 3.4.3 Treated tumor volume

Given that the treated tumor volume is substantially influenced by FUS, it is noteworthy that 8 studies still reported this outcome. These 8 studies encompassed 11 independent experiments. Meta-analysis of these 11 independent experiments demonstrated that combination therapy significantly reduced the treated tumor volume compared to immunotherapy alone (SMD = -1.68 [95% CI −2.35, −0.83]; *p* = 0.0001; **Figure 3E**). However, overall heterogeneity was high (*X²* = 33.03, *I²* = 73%).

### 3.5 Subgroup analysis

Considering the potential impacts of different immunotherapy agents, ultrasound treatment mechanisms, treatment sequences, tumor types, and transplantation sites on treatment outcomes, we performed further subgroup analyses (**Supplementary Figures S1**–**S5**). Immunotherapy agents were categorized into single and multiple agents (two or more combined immunotherapy drugs). Ultrasound treatment mechanisms were classified into mechanical and thermal effects (Kuai et al. (27) was excluded due to unclear mechanism). Combination treatment sequences were divided into FUS followed by immunotherapy and vice versa. Tumor models were classified by site (*in situ* vs. subcutaneous tumors) and type (breast cancer vs. other tumors).

#### 3.5.1 Heterogeneity of subgroup analysis

Subgroup analysis examined three primary outcomes (distant tumor volume, survival time, and treated tumor volume) considering various characteristics such as immunotherapeutic agents, ultrasound therapy mechanisms, treatment sequence, and tumor location and type. Immunotherapeutic agents contribute to heterogeneity, with the multi-drug subgroup achieving superior outcomes across all primary measures, and *I^2^* values reduced to 0% (**Supplementary Figure S1**). The sequence of ultrasound therapy, tumor location, and tumor type significantly influenced heterogeneity. Specifically, patients receiving FFUS followed by immunotherapy, those with *in situ* tumors, and the breast cancer subgroup experienced improved survival outcomes, with *I^2^* values reduced to 0% (**Supplementary Figures S3B**, **S4B**, **S5B**).

#### 3.5.2 Subgroup analysis results

Subgroup analysis provided insights into the benefits of combination therapy from different perspectives. The total results of the subgroup analyses are summarized in the **Graphical Abstract**. For immunotherapy agents, both single and multiple-agent groups showed the superiority of combination therapy in survival time and distant tumor volumes (**Supplementary Figure S1**). Subgroup analysis of ultrasound mechanisms indicated that although thermal and mechanical effects had similar effects on reducing tumor volume (**Supplementary Figure S2**), they differed significantly in their impact on survival time. In the thermal-effect subgroup, combination therapy did not provide any survival benefit over monotherapy (HR=0.75 [0.26, 2.19]; *p*=0.60). Conversely, the mechanical effect significantly improved survival time in mice (HR = 0.14 [0.06, 0.34]; *p* < 0.0001; **Supplementary Figure S2B**).

Similarly, altering the sequence of combination therapies significantly influenced survival time but did not affect their efficacy on tumor volume (**Supplementary Figure S3**).The group receiving immunotherapy first showed borderline statistical survival benefit over that receiving immunotherapy alone (HR=0.40 [0.16, 1.01]; *p*=0.05), whereas that receiving ultrasound first did show a significant difference (HR=0.18 [0.07, 0.51]; *p*=0.001) (**Supplementary Figure S3B**).Although tumor transplantation sites were on the body surface, considering the orthotopic sites of breast cancer and melanoma, we conducted subgroup analysis based on the transplantation sites. In terms of survival time, distant, and treated tumor volumes, both transplantation models showed consistent therapeutic benefits in the combination group (**Supplementary Figure S4**). Tumor type subgroup analysis revealed no significant differences in survival time and tumor volume between breast cancer and other tumor types in the combination group (**Supplementary Figure S5**).

### 3.6 Abscopal effect induced by FUS combined with immunotherapy on immune microenvironment

The therapeutic effects and changes in the immune microenvironment of each included study are summarized in **Table 2**. Combination therapy effectively reduced abscopal tumor volume in 11 of 12 studies, reduced treated tumor volume in 11 studies, and increased survival in7 studies. Significant increases in the number of anti-tumor immune cells, such as natural killer cells (NK), anti-tumor T cells, and M1 macrophages, were observed in the immune microenvironments of treated tumors, spleen, and distant tumors.

**Table 2:** Structure, target, and mechanism of potential inhibitors of NLRP3 inflammasome.

Author	Country	Year	Effect	Immune response mechanism
Michael Chavez	USA	2018	distance tumor volume▼	distance tumor: T cell, CD4^+^T cell, CD8^+^Tcell, IFNy^+^CD8^+^Tcell, F4/80^+^CD11b^+^CD45^+^cell▲ treated local tumor: F4/80^+^CD11b^+^CD45^+^ cell▲ spleen: CD169^+^macrophage cell▲ serum: CD169^+^macrophage cell, IFN-α, IFN-β▲ draining lymph node of distance tumor: CD169^+^cell, SIINFEKL^+^CD169^+^cell,SIINFEKL^+^CD169^+^MHCII^+^CD11C^+^cell,SIINFEKL^+^F4/80^+^MHCII^+^CD11b^+^cell▲
Jiawei Tang	China	2023	survival rate▲; distance tumor volume▼, treated local tumor volume▼	treated local tumor: CD8^+^T cell, TNFα^+^CD8^+^T cell, IFN-γ^+^CD8^+^T cell, Ki67^+^CD8^+^T cell, Ki67^+^CD4^+^cell▲ distance tumor: CD8^+^T cell▲, FOXP3^+^Treg ▼;
Matthew T. Silvestrini	USA	2017	survival rate▲; distance tumor volume▼, treated local tumor volume▼	treated local tumor: CD45^+^cell, CD3^+^T cell, CD4^+^T cell, CD8^+^Tcell, IFN-γ^+^CD8^+^Tcell, PD-L1^+^CD45^-^cell▲; PD-L1^+^CD45^+^ cell▼; M2 macrophage fraction▼; distance tumor: CD45^+^cell, IFN-γ^+^CD8^+^Tcell, M1 macrophage fraction▲; MDSCs cell▼; PD-L1^+^CD45^+^ cell▼; spleen: CD3^+^T cell, CD4^+^T cell, CD8^+^Tcell, IFN-γ^+^CD8^+^Tcell▲
James Wang	USA	2022	survival rate▲; distance tumor volume▼, treated local tumor volume▼	treated local tumor: CD4^+^Tcell, DC cell▲ distance tumor: CD4^+^Tcell, DC cell▲
Avinash Eranki	USA	2020	survival rate▲; distance tumor volume▼, treated local tumor volume▼	N/A
Shi Bin Qu	USA	2020	treated local tumor volume, distance tumor volume▼	treated local tumor: tumor infiltrating lymphocyte, GranzymeB^+^CD8^+^Tcell, PD-1^-^GranzymeB^+^CD8^+^Tcell, M1 macrophage▲; spleen: IFN-γ^+^CD4^+^Tcell, IL-2^+^CD8^+^Tcell, M1 macrophage▲; M2 macrophage fraction▼;
Mohit Pratap Singh	USA	2021	treated local tumor volume, distance tumor volume▼	distance tumor: CD8^+^Tcell▲;
Shinya Abe	USA	2022	treated local tumor volume, distance tumor volume▼	distance tumor: CD8^+^GranzymeB^+^cell, CD4^+^Tcell▲; treated local tumor: CD45^+^cell, CD69^+^CD4^+^Tcell, ICOS^+^CD4^+^Tcell, CD69^+^CD8^+^Tcell, ICOS^+^CD8^+^Tcell, CD8^+^GranzymeB^+^cell▲;
Mohit Pratap Singh	USA	2019	survival rate▲; distance tumor volume▼, treated local tumor volume▼	treated local tumor: CD3^+^Tcell▲ distance tumor: CD3^+^Tcell▲
Ting-Chuan Li	China	2020	survival rate▲; distance tumor volume▼, treated local tumor volume▼	treated local tumor: IFNγ^+^ CD4 T cell, IFNγ^+^ CD8 T cell, NK cell▲ distance tumor: IFNγ^+^ CD4 T cell, IFNγ^+^ CD8 T cells, NK cell▲
Xinping Kuai	China	2022	distance tumor volume▼ treated local tumor volume▼	treated local tumor: CD4^+^T cell, CD8^+^Tcell, DC cell▲
Jingnan Li	China	2022	survival rate▲; distance tumor volume▼, treated local tumor volume▼	distance tumor: CD4^+^ CD8^+^T cell▲; Tregs cell▼; treated local tumor: CD4^+^ CD8^+^T cell▲; Tregs cell▼;

N/A, Not Applicable.

### 3.7 Sensitivity analysis and publication bias

Visual inspection of funnel plot for survival time did not show any significant publication bias (**Figure 3B**), as the effect size distribution was largely symmetrical, and the Egger test (*p*= 0.798) suggested high robustness. However, the Egger tests for treated and distant tumor volumes were statistically significant (*p*= 0.007 and *p* = 0.042, respectively), indicating potential publication bias (**Figures 3D**, **F**). Therefore, sensitivity analysis using the trim-and-fill method was conducted to assess publication bias, and confirmed that the pooled results remained robust.

## 4 Discussion

**Figure 4:**
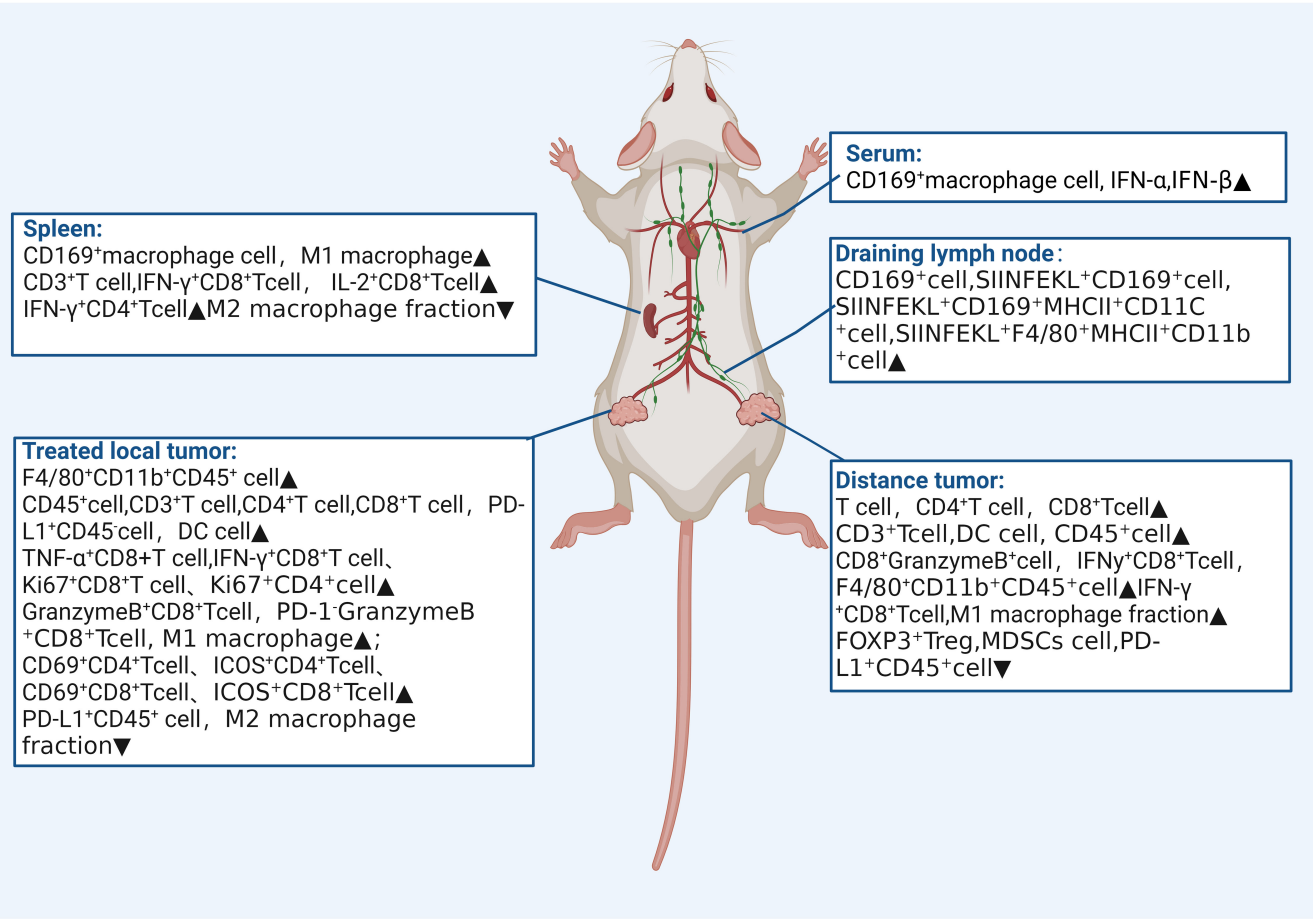
Impact on Immune microenvironment of focused ultrasound combined with immunotherapy in the spleen, serum, therapeutic draining lymph nodes, treated tumors, and distant tumors.

The abscopal effect, a promising phenomenon closely linked to immune activation, occurs as a synergistic therapeutic effect when localized treatment is combined with systemic immunotherapy. We conducted a systematic review and meta-analysis to evaluate the abscopal effect and its immune microenvironment influence in solid tumor animal models treated with Uncombined with immunotherapy. All included studies reported positive therapeutic effects of this combination therapy on mice. Following combined treatment, improvements were observed in distant tumor volume, survival time, and treated tumor volume. Additionally, the studies elucidated the effect of the immune microenvironment by which FUS combined with immunotherapy induces the abscopal effect (**Figure 4**). To our knowledge, this is the first meta-analysis to assess the abscopal effect of FUS combined with immunotherapy in solid tumor animal models.

FUS is a non-invasive, image-guided local tumor treatment technique. Under the monitoring of ultrasound or Magnetic resonance imaging, a focused transducer external to the body concentrates ultrasound energy to a focal point within the tissue, akin to an “invisible ultrasound scalpel,” enabling precise cutting treatment. FUS is an important supplement or alternative to traditional cancer treatments such as surgery, chemotherapy, and radiotherapy. It can modulate the tumor immune microenvironment, transforming immunologically “cold” tumors into reactive “hot” ones, and enhancing anti-tumor responses when combined with immunotherapy (29, 30). Among the 12 included studies, eight reported that FUS increased the number of activated T cells within the treated tumor microenvironment, with upregulation of CD8^+^ T cells and increased expression of IFN-γ and granzyme B. IFN-γ is closely related to transcription factor regulation, macrophage activation, and expression of antigen processing and presentation molecules, promoting ferroptosis in tumor cells and enhancing the cytotoxic activity of CD8^+^ T cells (31). Moreover, this immune-enhancing effect is not only limited to the treated site but also extends to distant tumors.

Moreover, exploration of the immune microenvironment in distant tumors revealed a significant increase in the number of T cells following combination therapy, accompanied by a decrease in that of myeloid-derived suppressor cells (MDSCs) and an increase in M1-type macrophage polarization. This suggests the systemic mobilization and activation of anti-tumor immunity. Originating from the bone marrow, MDSCs promote tumor escape by generating immunosuppressive cytokines, inducing regulatory T cells, and degrading key amino acids essential for T cell responses (32, 33). Additionally, MDSCs interfere with natural killer cell functions, altering the tumor microenvironment to support tumor growth and metastasis (34). Combination therapy effectively triggers the release of damage-associated molecular patterns (DAMPs) from injured and dying cells. Following treatment, calreticulin exposure on the cell membrane peaked at 6 h, and the levels of ATP and HMGB1 increased significantly (27, 35), indicating that combination therapy induces immunogenic cell death, thereby inhibiting tumor recurrence and metastasis.

Subgroup analyses provided several intriguing insights. First, the differing mechanisms of action of FUS have been a focal point of interest. The distinct mechanisms of thermal and mechanical effects are believed to elicit varying immunological responses. The thermal effect elevates the temperature of the targeted tissue (up to 60°C), leading to coagulative necrosis. Meanwhile, the mechanical effect disrupts cells, causing rupture and necrosis (36), and generates a cellular homogenate that releases more tissue debris, which is thought to induce a more robust immune response, thereby enhancing antitumor efficacy (24, 37). Our results reinforce that combination therapy in the mechanical-effect group offers a clear survival advantage over immunotherapy alone, a benefit not observed with the thermal effect. This distinction is attributed to the unique mechanism of action in mechanical ablation. Unlike thermal effects, which rely on heat, mechanical effects employ acoustic cavitation to physically disrupt tumor cells and their internal structures, leading to cell membrane rupture and death. This mechanical disruption generates a broader spectrum of tumor-associated antigens and damage-associated molecular patterns DAMPs, which effectively recruit and activate antigen-presenting cells such as dendritic cells and macrophages. Subsequently, this stimulates a more robust anti-tumor immune response, particularly through the infiltration of cytotoxic T cells into the tumor microenvironment (38, 39). Nevertheless, the limited number of studies included in the survival outcome analysis necessitates a cautious interpretation of these findings.

The sequence of FUS and immunotherapy administration was also explored. Silvestrini et al. found that the order of these treatments affects the immune efficacy of the combination therapy (25). FUS can break down tumor tissue into a rich mixture of DAMPs, which are recognized by Toll-like and Nod-like receptors, thus activating anti-tumor immunity (12, 40). Pre-administration of immunotherapeutic agents can create a pre-activated immune microenvironment, which promptly recognizes DAMPs post-ultrasound treatment, thereby initiating immune activation. However, our results presented a contrasting conclusion regarding the treatment sequence. The survival benefit of immunotherapy followed by FUS showed borderline statistical significance, indicating no substantial difference attributable to the treatment sequence. We attribute this to the fact in three of the included studies (23, 25, 28), the survival curves of the combination and immunotherapy groups did not show statistical differences, although there was significant difference in tumor volume. Similar to the previous subgroup analysis of ultrasound effects, we advise caution in interpreting this conclusion. In other subgroups, such as different numbers of immunotherapeutic agents, tumor types, and tumor transplantation sites, the combination therapy group consistently demonstrated improved efficacy. This consistency suggests that FUS combined with immunotherapy can exert abscopal effects across various tumors, treatments, and models.

In this meta-analysis, we observed substantial heterogeneity in the measurement of treated tumor growth volume, even when using a random-effects model. Subgroup analyses suggest that this heterogeneity may stem from the different types of immunotherapeutic agents and tumor models utilized. The use of single immunotherapeutic agents and various tumor models contributed to the observed heterogeneity. Therefore, further analysis of the specific types of drugs or tumors might help elucidate the sources of this heterogeneity. However, owing to the limited sample size of the included studies, additional experiments are required to explore these variations. Nevertheless, sensitivity analysis confirmed the robustness of our findings. Despite performing an extensive literature search, we cannot exclude the possibility that some studies, such as conference abstracts and supplements, were unavailable or that some negative results remain unpublished. Given that results from animal experiments cannot fully replicate the complex pathophysiology of clinical settings, large-scale clinical trials are required to validate these findings before clinical extrapolation.

This study has some limitations. First, despite our efforts to minimize potential biases, the limited number of experiments and heterogeneity may preclude strong evidence from subgroup and immune environment analyses. Therefore, the results of these analyses should be interpreted with utmost caution. Second, our study focused primarily on the thermal and mechanical effects of FUS combined with immunotherapeutic agents, and excluded emerging techniques such as sonodynamic therapy, which may limit the generalizability of our conclusions. Finally, the limitations of the animal models and immunotherapeutic agents included necessitate further diverse studies to confirm these results.

In summary, this systematic review and meta-analysis of preclinical animal experiments evaluated the abscopal effect of combined FUS and immunotherapy in mouse models of solid tumors. FUS can modulate the tumor immune microenvironment and enhance anti-tumor immunity, demonstrating an abscopal effect and improving overall survival when combined with immunotherapeutic agents. These findings provide deeper insights into the synergistic effects of FUS and immunotherapy, promoting their combined use in clinical applications and offering a new therapeutic paradigm for treating advanced tumors.

## Data Availability

The original contributions presented in the study are included in the article/**Supplementary Material**. Further inquiries can be directed to the corresponding authors.
