# Supplementary material for: Abscopal effect of focused ultrasound combined immunotherapy in animal solid tumor model: a systematic reviews and meta-analysis
Source: Front Immunol. 2024 Dec 13;15:1474343. doi: 10.3389/fimmu.2024.1474343 (PMC11671366; doi:10.3389/fimmu.2024.1474343)
Supplement: Supplementary file 1 [file DataSheet1.docx]

Supplementary Material

# Supplementary Figures and Tables

## Supplementary Tables

**Supplementary Table 1.** Search strategy.

| Database | Search terms | Results |
| --- | --- | --- |
| **Pubmed** |  | **167 results** |
| #1 | "Immunotherapy"[Mesh] OR "Antineoplastic Agents, Immunological"[Mesh] OR "Immune Checkpoint Inhibitors"[Mesh] OR "Immunomodulating Agents"[Mesh] OR "Toll-Like Receptor Agonists"[Mesh] OR "Immune Checkpoint Proteins"[Mesh] | 398,142 |
| #2 | (Checkpoint Inhibitors, Immune*[Title/Abstract]) OR (Immune Checkpoint Inhibitor*[Title/Abstract]) OR (Checkpoint Inhibitor, Immune*[Title/Abstract]) OR (Immune Checkpoint Blockers*[Title/Abstract]) OR (Immunotherapies*[Title/Abstract]) OR (Checkpoint Blockers, Immune*[Title/Abstract]) OR (Immune Checkpoint Blockade*[Title/Abstract]) OR (Checkpoint Blockade, Immune*[Title/Abstract]) OR (Immune Checkpoint Inhibition*[Title/Abstract]) OR (Checkpoint Inhibition, Immune*[Title/Abstract]) OR (PD-L1 Inhibitors*[Title/Abstract]) OR (PD L1 Inhibitors*[Title/Abstract]) OR (PD-L1 Inhibitor*[Title/Abstract]) OR (PD L1 Inhibitor*[Title/Abstract]) OR (Programmed Death-Ligand 1 Inhibitors*[Title/Abstract]) OR (Programmed Death Ligand 1 Inhibitors*[Title/Abstract]) OR (PD-1-PD-L1 Blockade*[Title/Abstract]) OR (Blockade, PD-1-PD-L1*[Title/Abstract]) OR (PD 1 PD L1 Blockade*[Title/Abstract]) OR (CTLA-4 Inhibitors*[Title/Abstract]) OR (CTLA 4 Inhibitors*[Title/Abstract]) OR (CTLA-4 Inhibitor*[Title/Abstract]) OR (CTLA 4 Inhibitor*[Title/Abstract]) OR (Cytotoxic T-Lymphocyte-Associated Protein 4 Inhibitors*[Title/Abstract]) OR (Cytotoxic T Lymphocyte Associated Protein 4 Inhibitors*[Title/Abstract]) OR (Cytotoxic T-Lymphocyte-Associated Protein 4 Inhibitor*[Title/Abstract]) OR (Cytotoxic T Lymphocyte Associated Protein 4 Inhibitor*[Title/Abstract]) OR (PD-1 Inhibitors*[Title/Abstract]) OR (PD 1 Inhibitors*[Title/Abstract]) OR (PD-1 Inhibitor*[Title/Abstract]) OR (Inhibitor, PD-1*[Title/Abstract]) OR (PD 1 Inhibitor*[Title/Abstract]) OR (Programmed Cell Death Protein 1 Inhibitor*[Title/Abstract]) OR (Programmed Cell Death Protein 1 Inhibitors*[Title/Abstract]) OR (Agents, Immunomodulating*[Title/Abstract]) OR (Immunomodulatory Compounds*[Title/Abstract]) OR (Immunomodulating Drugs*[Title/Abstract]) OR (Immunomodulatory Drugs*[Title/Abstract]) OR (Drugs, Immunomodulatory*[Title/Abstract]) OR (Immunomodulatory Agents*[Title/Abstract]) OR (Agents, Immunomodulatory*[Title/Abstract]) OR (Immunomodulatory IMiD Drugs*[Title/Abstract]) OR (Drugs, Immunomodulatory IMiD*[Title/Abstract]) OR (IMiDs*[Title/Abstract]) OR (Immunomodulatory IMiDE Drugs*[Title/Abstract]) OR (Drugs, Immunomodulatory IMiDE*[Title/Abstract]) OR (Agonists, Toll-Like Receptor*[Title/Abstract]) OR (Receptor Agonists, Toll-Like*[Title/Abstract]) OR (Toll Like Receptor Agonists*[Title/Abstract]) OR (Toll-Like Receptor Agonist*[Title/Abstract]) OR (Agonist, Toll-Like Receptor*[Title/Abstract]) OR (Receptor Agonist, Toll-Like*[Title/Abstract]) OR (Toll Like Receptor Agonist*[Title/Abstract]) OR (TLR Agonists*[Title/Abstract]) OR (Agonists, TLR*[Title/Abstract]) OR (Immune Checkpoint Molecules*[Title/Abstract]) OR (Immune Checkpoint Protein*[Title/Abstract]) OR (Checkpoint Protein, Immune*[Title/Abstract]) OR (Protein, Immune Checkpoint*[Title/Abstract]) OR (Immune Checkpoint Molecule*[Title/Abstract]) OR (Checkpoint Molecule, Immune*[Title/Abstract]) OR (Molecule, Immune Checkpoint*[Title/Abstract]) OR (Stimulatory Checkpoint Molecules*[Title/Abstract]) OR (Stimulatory Checkpoint Molecule*[Title/Abstract]) OR (Checkpoint Molecule, Stimulatory*[Title/Abstract]) OR (Inhibitory Checkpoint Molecules*[Title/Abstract]) OR (Inhibitory Checkpoint Molecule*[Title/Abstract]) OR (Checkpoint Molecule, Inhibitory*[Title/Abstract]) OR (antibody therapies*[Title/Abstract]) OR (CTLA-4 blockade*[Title/Abstract]) OR (agonistic antibody*[Title/Abstract]) OR (immunotherapy*[Title/Abstract]) OR (Antineoplastic Agents, Immunological*[Title/Abstract]) OR (Immune Checkpoint Inhibitors*[Title/Abstract]) OR (Immunomodulating Agents*[Title/Abstract]) OR (Toll-Like Receptor Agonists*[Title/Abstract]) OR (Immune Checkpoint Proteins*[Title/Abstract]) | 191,023 |
| #3 | "Ultrasonic Therapy"[Mesh] OR "Ultrasonic Surgical Procedures"[Mesh] OR "Ultrasound, High-Intensity Focused, Transrectal"[Mesh] OR “High-Intensity Focused Ultrasound Ablation"[Mesh] | 40,290 |
| #4 | (focus ultrasound*[Title/Abstract] ) OR (focus ultrasound surgery*[Title/Abstract]) OR (ultrasound ablation*[Title/Abstract]) OR (Ultrasonic Therapy*[Title/Abstract]) OR (histotripsy*[Title/Abstract]) OR (Ultrasonic Surgical Procedures*[Title/Abstract]) OR (Ultrasound, High-Intensity Focused, Transrectal*[Title/Abstract]) OR (Therapies, Ultrasonic*[Title/Abstract]) OR (Ultrasonic Therapies*[Title/Abstract]) OR (Therapeutic Ultrasound*[Title/Abstract]) OR (Ultrasound, Therapeutic*[Title/Abstract]) OR (Therapy, Ultrasonic*[Title/Abstract]) OR (Ultrasound Therapy*[Title/Abstract]) OR (Therapies, Ultrasound*[Title/Abstract]) OR (Therapy, Ultrasound*[Title/Abstract]) OR (Ultrasound Therapies*[Title/Abstract]) OR (Transrectal High-Intensity Focused Ultrasound*[Title/Abstract]) OR (Transrectal High Intensity Focused Ultrasound*[Title/Abstract]) OR (Surgical Procedure, Ultrasonic*[Title/Abstract]) OR (Surgical Procedures, Ultrasonic*[Title/Abstract]) OR (Ultrasonic Surgical Procedure*[Title/Abstract]) OR (Ultrasound Surgery*[Title/Abstract]) OR (Surgeries, Ultrasound*[Title/Abstract]) OR (Surgery, Ultrasound*[Title/Abstract]) OR (Ultrasound Surgeries*[Title/Abstract]) OR (Ultrasound Surgical Procedures*[Title/Abstract]) OR (Surgical Procedure, Ultrasound*[Title/Abstract]) OR (Surgical Procedures, Ultrasound*[Title/Abstract]) OR (Ultrasound Surgical Procedure*[Title/Abstract]) OR (Ultrasonic Surgery*[Title/Abstract]) OR (Surgeries, Ultrasonic*[Title/Abstract]) OR(Surgery, Ultrasonic*[Title/Abstract]) OR (Ultrasonic Surgeries*[Title/Abstract]) OR (image-guided thermal ablation*[Title/Abstract]) OR (focused ultrasound*[Title/Abstract]) OR (focused ultrasound tumor ablation*[Title/Abstract]) OR (focused ultrasound heating*[Title/Abstract]) OR (thermal ablation*[Title/Abstract]) OR (Mechanical ablation*[Title/Abstract]) | 47,234 |
| #5 | "Neoplasms"[Mesh] OR "Sarcoma"[Mesh]OR “Carcinoma"[Mesh] | 4,027,429 |
| #6 | Tumor* [Title/Abstract] OR Neoplasm* [Title/Abstract] OR Tumors* [Title/Abstract] OR Neoplasia* [Title/Abstract] OR Neoplasias* [Title/Abstract] OR Cancer* [Title/Abstract] OR Cancers* [Title/Abstract] OR Malignant Neoplasm* [Title/Abstract] OR Malignancy* [Title/Abstract] OR Malignancies* [Title/Abstract] OR Malignant Neoplasms* [Title/Abstract] OR Neoplasm, Malignant* [Title/Abstract] OR Neoplasms, Malignant* [Title/Abstract] OR melanoma* [Title/Abstract] OR Sarcomas* [Title/Abstract] OR Carcinoma* [Title/Abstract] OR Sarcomas* [Title/Abstract] OR Sarcoma, Soft Tissue* [Title/Abstract] OR Sarcomas, Soft Tissue* [Title/Abstract] OR Soft Tissue Sarcoma* [Title/Abstract] OR Soft Tissue Sarcomas* [Title/Abstract] OR Sarcoma, Epithelioid* [Title/Abstract] OR Epithelioid Sarcoma* [Title/Abstract] OR Epithelioid Sarcomas* [Title/Abstract] OR Sarcomas, Epithelioid* [Title/Abstract] OR Sarcoma, Spindle Cell* [Title/Abstract] OR Sarcomas, Spindle Cell* [Title/Abstract] OR Spindle Cell Sarcoma* [Title/Abstract] OR Spindle Cell Sarcomas* [Title/Abstract] OR Carcinomas* [Title/Abstract] OR Epithelial Neoplasms, Malignant* [Title/Abstract] OR Malignant Epithelial Neoplasms* [Title/Abstract] OR Epithelial Neoplasm, Malignant* [Title/Abstract] OR Malignant Epithelial Neoplasm* [Title/Abstract] OR Neoplasm, Malignant Epithelial* [Title/Abstract] OR Neoplasms, Malignant Epithelial* [Title/Abstract] OR Epithelial Tumors, Malignant* [Title/Abstract] OR Epithelial Tumor, Malignant* [Title/Abstract] OR Malignant Epithelial Tumor* [Title/Abstract] OR Malignant Epithelial Tumors* [Title/Abstract] OR Tumor, Malignant Epithelial* [Title/Abstract] OR Epithelioma* [Title/Abstract] OR Epitheliomas* [Title/Abstract] OR Carcinoma, Undifferentiated* [Title/Abstract] OR Undifferentiated Carcinoma* [Title/Abstract] OR Undifferentiated Carcinomas* [Title/Abstract] OR Carcinoma, Anaplastic* [Title/Abstract] OR Anaplastic Carcinoma* [Title/Abstract] OR Anaplastic Carcinomas* [Title/Abstract] OR Carcinoma, Spindle-Cell* [Title/Abstract] OR Carcinoma, Spindle Cell* [Title/Abstract] OR Spindle-Cell Carcinoma* [Title/Abstract] OR Spindle-Cell Carcinomas* [Title/Abstract] OR Carcinomatosis* [Title/Abstract] OR Carcinomatoses* [Title/Abstract] OR melanoma* [Title/Abstract] | 4,083,132 |
| #7 | #1 OR #2 | 502,665 |
| #8 | #3 OR #4 | 78,169 |
| #9 | #5 OR #6 | 5,273,475 |
| #10 | #7 AND #8 AND #9 AND ((animal[Filter]) AND (2000/1/1:2024/10/14[pdat])) | 167 |
| **Embase** |  | **484 results** |
| #1 | 'immunotherapy'/exp OR 'immunological antineoplastic agent'/exp OR 'immune checkpoint inhibitor'/exp OR 'immunomodulating agent'/exp OR 'toll like receptor agonist'/exp OR 'immune checkpoint protein'/exp | 2,714,528 |
| #2 | ‘Checkpoint Inhibitors, Immune’:ti,ab OR ‘Immune Checkpoint Inhibitor’:ti,ab OR ‘Checkpoint Inhibitor, Immune’:ti,ab OR ‘Immune Checkpoint Blockers’:ti,ab OR ‘Immunotherapies’:ti,ab OR ‘Checkpoint Blockers, Immune’:ti,ab OR ‘Immune Checkpoint Blockade’:ti,ab OR ‘Checkpoint Blockade, Immune’:ti,ab OR ‘Immune Checkpoint Inhibition’:ti,ab OR ‘Checkpoint Inhibition, Immune’:ti,ab OR ‘PD-L1 Inhibitors’:ti,ab OR ‘PD L1 Inhibitors’:ti,ab OR ‘PD-L1 Inhibitor’:ti,ab OR ‘PD L1 Inhibitor’:ti,ab OR ‘Programmed Death-Ligand 1 Inhibitors’:ti,ab OR ‘Programmed Death Ligand 1 Inhibitors’:ti,ab OR ‘PD-1-PD-L1 Blockade’:ti,ab OR ‘Blockade, PD-1-PD-L1’:ti,ab OR ‘PD 1 PD L1 Blockade’:ti,ab OR ‘CTLA-4 Inhibitors’:ti,ab OR ‘CTLA 4 Inhibitors’:ti,ab OR ‘CTLA-4 Inhibitor’:ti,ab OR ‘CTLA 4 Inhibitor’:ti,ab OR ‘Cytotoxic T-Lymphocyte-Associated Protein 4 Inhibitors’:ti,ab OR ‘Cytotoxic T Lymphocyte Associated Protein 4 Inhibitors’:ti,ab OR ‘Cytotoxic T-Lymphocyte-Associated Protein 4 Inhibitor’:ti,ab OR ‘Cytotoxic T Lymphocyte Associated Protein 4 Inhibitor’:ti,ab OR ‘PD-1 Inhibitors’:ti,ab OR ‘PD 1 Inhibitors’:ti,ab OR ‘PD-1 Inhibitor’:ti,ab OR ‘Inhibitor, PD-1’:ti,ab OR ‘PD 1 Inhibitor’:ti,ab OR ‘Programmed Cell Death Protein 1 Inhibitor’:ti,ab OR ‘Programmed Cell Death Protein 1 Inhibitors’:ti,ab OR ‘Agents, Immunomodulating’:ti,ab OR ‘Immunomodulatory Compounds’:ti,ab OR ‘Immunomodulating Drugs’:ti,ab OR ‘Immunomodulatory Drugs’:ti,ab OR ‘Drugs, Immunomodulatory’:ti,ab OR ‘Immunomodulatory Agents’:ti,ab OR ‘Agents, Immunomodulatory’:ti,ab OR ‘Immunomodulatory IMiD Drugs’:ti,ab OR ‘Drugs, Immunomodulatory IMiD’:ti,ab OR ‘IMiDs’:ti,ab OR ‘Immunomodulatory IMiDE Drugs’:ti,ab OR ‘Drugs, Immunomodulatory IMiDE’:ti,ab OR ‘Agonists, Toll-Like Receptor’:ti,ab OR ‘Receptor Agonists, Toll-Like’:ti,ab OR ‘Toll Like Receptor Agonists’:ti,ab OR ‘Toll-Like Receptor Agonist’:ti,ab OR ‘Agonist, Toll-Like Receptor’:ti,ab OR ‘Receptor Agonist, Toll-Like’:ti,ab OR ‘Toll Like Receptor Agonist’:ti,ab OR ‘TLR Agonists’:ti,ab OR ‘Agonists, TLR’:ti,ab OR ‘Immune Checkpoint Molecules’:ti,ab OR ‘Immune Checkpoint Protein’:ti,ab OR ‘Checkpoint Protein, Immune’:ti,ab OR ‘Protein, Immune Checkpoint’:ti,ab OR ‘Immune Checkpoint Molecule’:ti,ab OR ‘Checkpoint Molecule, Immune’:ti,ab OR ‘Molecule, Immune Checkpoint’:ti,ab OR ‘Stimulatory Checkpoint Molecules’:ti,ab OR ‘Stimulatory Checkpoint Molecule’:ti,ab OR ‘Checkpoint Molecule, Stimulatory’:ti,ab OR ‘Inhibitory Checkpoint Molecules’:ti,ab OR ‘Inhibitory Checkpoint Molecule’:ti,ab OR ‘Checkpoint Molecule, Inhibitory’:ti,ab OR ‘antibody therapies’:ti,ab OR ‘CTLA-4 blockade’:ti,ab OR ‘agonistic antibody’:ti,ab OR ‘immunotherapy’:ti,ab OR ‘Antineoplastic Agents, Immunological’:ti,ab OR ‘Immune Checkpoint Inhibitors’:ti,ab OR ‘Immunomodulating Agents’:ti,ab OR ‘Toll-Like Receptor Agonists’:ti,ab OR ‘Immune Checkpoint Proteins’:ti,ab | 270,490 |
| #3 | 'ultrasound therapy'/exp OR 'ultrasound therapy' OR 'ultrasound surgery'/exp OR 'ultrasound surgery' OR 'transrectal high intensity focused ultrasound'/exp OR 'transrectal high intensity focused ultrasound' OR 'high intensity ultrasound'/exp OR 'high intensity ultrasound' | 32,527 |
| #4 | 'focus ultrasound':ti,ab OR 'focus ultrasound surgery':ti,ab OR 'ultrasound ablation':ti,ab OR 'ultrasonic therapy':ti,ab OR 'histotripsy':ti,ab OR 'ultrasonic surgical procedures':ti,ab OR 'ultrasound, high-intensity focused, transrectal':ti,ab OR 'therapies, ultrasonic':ti,ab OR 'ultrasonic therapies':ti,ab OR 'therapeutic ultrasound':ti,ab OR 'ultrasound, therapeutic':ti,ab OR 'therapy, ultrasonic':ti,ab OR 'ultrasound therapy':ti,ab OR 'therapies, ultrasound':ti,ab OR 'therapy, ultrasound':ti,ab OR 'ultrasound therapies':ti,ab OR 'transrectal high-intensity focused ultrasound':ti,ab OR 'transrectal high intensity focused ultrasound':ti,ab OR 'surgical procedure, ultrasonic':ti,ab OR 'surgical procedures, ultrasonic':ti,ab OR 'ultrasonic surgical procedure':ti,ab OR 'ultrasound surgery':ti,ab OR 'surgeries, ultrasound':ti,ab OR 'surgery, ultrasound':ti,ab OR 'ultrasound surgeries':ti,ab OR 'ultrasound surgical procedures':ti,ab OR 'surgical procedure, ultrasound':ti,ab OR 'surgical procedures, ultrasound':ti,ab OR 'ultrasound surgical procedure':ti,ab OR 'ultrasonic surgery':ti,ab OR 'surgeries, ultrasonic':ti,ab OR 'surgery, ultrasonic':ti,ab OR 'ultrasonic surgeries':ti,ab OR 'image-guided thermal ablation':ti,ab OR 'focused ultrasound':ti,ab OR 'focused ultrasound tumor ablation':ti,ab OR 'focused ultrasound heating':ti,ab OR 'thermal ablation':ti,ab OR 'mechanical ablation':ti,ab | 22,201 |
| #5 | 'neoplasm'/exp OR 'neoplasm' OR 'sarcoma'/exp OR 'sarcoma' OR 'carcinoma'/exp OR 'carcinoma' | 6,521,400 |
| #6 | 'tumor':ti,ab OR 'neoplasm':ti,ab OR 'tumors':ti,ab OR 'neoplasia':ti,ab OR 'neoplasias':ti,ab OR 'cancer':ti,ab OR 'cancers':ti,ab OR 'malignant neoplasm':ti,ab OR 'malignancy':ti,ab OR 'malignancies':ti,ab OR 'malignant neoplasms':ti,ab OR 'neoplasm, malignant':ti,ab OR 'neoplasms, malignant':ti,ab OR 'carcinoma':ti,ab OR 'sarcomas':ti,ab OR 'sarcoma, soft tissue':ti,ab OR 'sarcomas, soft tissue':ti,ab OR 'soft tissue sarcoma':ti,ab OR 'soft tissue sarcomas':ti,ab OR 'sarcoma, epithelioid':ti,ab OR 'epithelioid sarcoma':ti,ab OR 'epithelioid sarcomas':ti,ab OR 'sarcomas, epithelioid':ti,ab OR 'sarcoma, spindle cell':ti,ab OR 'sarcomas, spindle cell':ti,ab OR 'spindle cell sarcoma':ti,ab OR 'spindle cell sarcomas':ti,ab OR 'carcinomas':ti,ab OR 'epithelial neoplasms, malignant':ti,ab OR 'malignant epithelial neoplasms':ti,ab OR 'epithelial neoplasm, malignant':ti,ab OR 'malignant epithelial neoplasm':ti,ab OR 'neoplasm, malignant epithelial':ti,ab OR 'neoplasms, malignant epithelial':ti,ab OR 'epithelial tumors, malignant':ti,ab OR 'epithelial tumor, malignant':ti,ab OR 'malignant epithelial tumor':ti,ab OR 'malignant epithelial tumors':ti,ab OR 'tumor, malignant epithelial':ti,ab OR 'epithelioma':ti,ab OR 'epitheliomas':ti,ab OR 'carcinoma, undifferentiated':ti,ab OR 'undifferentiated carcinoma':ti,ab OR 'undifferentiated carcinomas':ti,ab OR 'carcinoma, anaplastic':ti,ab OR 'anaplastic carcinoma':ti,ab OR 'anaplastic carcinomas':ti,ab OR 'carcinoma, spindle-cell':ti,ab OR 'carcinoma, spindle cell':ti,ab OR 'spindle-cell carcinoma':ti,ab OR 'spindle-cell carcinomas':ti,ab OR 'carcinomatosis':ti,ab OR 'carcinomatoses':ti,ab OR 'melanoma':ti,ab | 5,443,273 |
| #7 | #1 OR#2 | 2,762,032 |
| #8 | #3 OR #4 | 43,398 |
| #9 | #5 OR #6 | 7,426,215 |
| #10 | #7 AND #8 AND #9 AND [animals]/lim | 1,720 |
| #11 | #7 AND #8 AND #9 AND [animals]/lim AND [01-01-2001]/sd NOT [14-10-2024]/sd | 484 |
| **Web of Science** |  | **157 results** |
| #1 | TS=(Immunotherapy* OR Antineoplastic Agents, Immunological* OR Immune Checkpoint Inhibitors* OR Immunomodulating Agents* OR Toll-Like Receptor Agonists* OR Immune Checkpoint Proteins* OR Checkpoint Inhibitors, Immune* OR Immune Checkpoint Inhibitor* OR Checkpoint Inhibitor, Immune* OR Immune Checkpoint Blockers* OR Immunotherapies* OR Checkpoint Blockers, Immune* OR Immune Checkpoint Blockade* OR Checkpoint Blockade, Immune* OR Immune Checkpoint Inhibition* OR Checkpoint Inhibition, Immune* OR PD-L1 Inhibitors* OR PD L1 Inhibit ors* OR PD-L1 Inhibitor* OR PD L1 Inhibitor* OR Programmed Death-Ligand 1 Inhibit ors* OR Programmed Death Ligand 1 Inhibitors* OR PD-1-PD-L1 Blockade* OR Blockade, PD-1-PD-L1* OR PD 1 PD L1 Blockade* OR CTLA-4 Inhibitors* OR CTLA 4 Inhibitors* OR CTLA-4 Inhibitor* OR CTLA 4 Inhibitor* OR Cytotoxic T-Lymphocyte-Associated Protein 4 Inhibitors* OR Cytotoxic T Lymphocyte Associated Protein 4 Inhibitors* OR Cytotoxic T-Lymphocyte-Associated Protein 4 Inhibitor* OR Cytotoxic T Lymphocyte Associated Protein 4 Inhibitor* OR PD-1 Inhibitors* OR PD 1 Inhibitors* OR PD-1 Inhibitor* OR Inhibitor, PD-1* OR PD 1 Inhibitor* OR Programmed Cell Death Protein 1 Inhibit Inhibitor * OR Programmed Cell Death Protein 1 Inhibitors* OR Agents, Immunomodulating* OR Immunomodulatory Compounds* OR Immunomodulating Drugs* OR Immunomodulatory Drugs* OR Drugs, Immunomodulatory* OR Immunomodulatory Agents* OR Agents, Immunomodulatory* OR Immunomodulatory IMiD Drugs* OR Drugs, Immunomodulatory IMiD* OR IMiDs* OR Immunomodulatory IMiDE Drugs* OR Drugs, Immunomodulatory IMiDE* OR Agonists, Toll-Like Receptor* OR Receptor Agonists, Toll-Like* OR Toll Like Receptor Agonists* OR Toll-Like Receptor Agonist* OR Agonist, Toll-Like Receptor* OR Receptor Agonist, Toll-Like* OR Toll Like Receptor Agonist* OR TLR Agonists* OR Agonists, TLR* OR Immune Checkpoint Molecules* OR Immune Checkpoint Protein* OR Checkpoint Protein, Immune* OR Protein, Immune Checkpoint* OR Immune Checkpoint Molecule* OR Checkpoint Molecule, Immune* OR Molecule, Immune Checkpoint* OR Stimulatory Checkpoint Molecules* OR Stimulatory Checkpoint Molecule* OR Checkpoint Molecule, Stimulatory* OR Inhibitory Checkpoint Molecules* OR Inhibitory Checkpoint Molecule* OR Checkpoint Molecule, Inhibitory* OR antibody therapies* OR CTLA-4 blockade* OR agonistic antibody* OR immunotherapy* OR Antineoplastic Agents, Immunological* OR Immune Checkpoint Inhibitors* OR Immunomodulating Agents* OR Toll-Like Receptor Agonists* OR Immune Checkpoint Proteins*) | 306,246 |
| #2 | TS=(Ultrasonic Therapy* OR Ultrasonic Surgical Procedures* OR Ultrasound, High-Intensity Focused, Transrectal* OR High-Intensity Focused Ultrasound Ablation * OR focus ultrasound* OR focus ultrasound surgery* OR ultrasound ablation* OR Ultrasonic Therapy* OR histotripsy* OR Ultrasonic Surgical Procedures* OR Ultrasound, High-Intensity Focused, Transrectal* OR Therapies, Ultrasonic* OR Ultrasonic Therapies* OR Therapeutic Ultrasound* OR Ultrasound, Therapeutic* OR Therapy, Ultrasonic* OR Ultrasound Therapy* OR Therapies, Ultrasound* OR Therapy, Ultrasound* OR Ultrasound Therapies* OR Transrectal High-Intensity Focused Ultrasound* OR Transrectal High Intensity Focused Ultrasound* OR Surgical Procedure, Ultrasonic* OR Surgical Procedures, Ultrasonic* OR Ultrasonic Surgical Procedure* OR Ultrasound Surgery* OR Surgeries, Ultrasound* OR Surgery, Ultrasound* OR Ultrasound Surgeries* OR Ultrasound Surgical Procedures* OR Surgical Procedure, Ultrasound* OR Surgical Procedures, Ultrasound* OR Ultrasound Surgical Procedure* OR Ultrasonic Surgery* OR Surgeries, Ultrasonic* OR Surgery, Ultrasonic* OR Ultrasonic Surgeries* OR image-guided thermal ablation* OR focused ultrasound* OR focused ultrasound tum OR ablation* OR focused ultrasound heating* OR thermal ablation* OR Mechanical ablation*) | 368,211 |
| #3 | TS=(Neoplasms* OR Sarcoma* OR Carcinoma* OR Tumor* OR Neoplasm* OR Tumors* OR Neoplasia* OR Neoplasias* OR Cancer* OR Cancers* OR Malignant Neoplasm*OR Malignancy* OR Malignancies* OR Malignant Neoplasms* OR Neoplasm, Malignant* OR Neoplasms, Malignant* OR melanoma* OR Sarcomas* OR Carcinoma* OR Sarcomas* OR Sarcoma, Soft Tissue* OR Sarcomas, Soft Tissue* OR Soft Tissue Sarcoma* OR Soft Tissue Sarcomas* OR Sarcoma, Epithelioid* OR Epithelioid Sarcoma* OR Epithelioid Sarcomas* OR Sarcomas, Epithelioid* OR Sarcoma, Spindle Cell* OR Sarcomas, Spindle Cell* OR Spindle Cell Sarcoma* OR Spindle Cell Sarcomas* OR Carcinomas* OR Epithelial Neoplasms, Malignant* OR Malignant Epithelial Neoplasms* OR Epithelial Neoplasm, Malignant* OR Malignant Epithelial Neoplasm* OR Neoplasm, Malignant Epithelial* OR Neoplasms, Malignant Epithelial* OR Epithelial Tum ORs, Malignant* OR Epithelial Tumor, Malignant* OR Malignant Epithelial Tumor* OR Malignant Epithelial Tumors* OR Tumor, Malignant Epithelial* OR Epithelioma* OR Epitheliomas* OR Carcinoma, Undifferentiated* OR Undifferentiated Carcinoma* OR Undifferentiated Carcinomas* OR Carcinoma, Anaplastic* OR Anaplastic Carcinoma* OR Anaplastic Carcinomas* OR Carcinoma, Spindle-Cell* OR Carcinoma, Spindle Cell* OR Spindle-Cell Carcinoma* OR Spindle-Cell Carcinomas* OR Carcinomatosis* OR Carcinomatoses* OR melanoma*) | 5,101,725 |
| #4 | #1 AND #2 AND #3 AND TS=(animals) | 157 |
| **SinoMed** |  | **8 results** |
| #1 | "高强聚焦超声消融"[不加权:扩展] OR "体外冲击波疗法"[不加权:扩展] OR "体外冲击波疗法"[不加权:扩展] OR "体外冲击波疗法"[不加权:扩展] OR "体外冲击波疗法"[不加权:扩展] OR "聚焦超声” [全部字段:智能] OR “聚焦超声消融治疗” [全部字段:智能] OR “聚焦超声热疗” [全部字段:智能] OR “低强度聚焦超声+ 高强聚焦超声” [全部字段:智能] OR “聚焦超声消融术” [全部字段:智能] OR “高频聚焦超声” [全部字段:智能] OR “高强聚焦超声消融” [全部字段:智能] OR “'低强度聚焦超声(lifu)'“ [全部字段:智能] OR “合成孔径聚焦超声” [全部字段:智能] OR “相控聚焦超声” [全部字段:智能] OR “高强度体外聚焦超声” [全部字段:智能] OR “高功率聚焦超声” [全部字段:智能] OR “聚焦超声治疗” [全部字段:智能] OR “聚焦超声消融” [全部字段:智能] OR “聚焦超声刀” [全部字段:智能] OR “聚焦超声波” [全部字段:智能] OR “聚焦超声技术” [全部字段:智能] OR “高强聚焦超声消融” [全部字段:智能] OR “聚焦超声手术” [全部字段:智能] OR “聚焦超声热疗” [全部字段:智能] OR “体外高强度聚焦超声疗法” [全部字段:智能] OR “体外高强度聚焦超声治疗” [全部字段:智能] OR “高强度聚焦超声疗法” [全部字段:智能] OR “高强度聚焦超声治疗” [全部字段:智能] | 6183 |
| #2 | "免疫检查点抑制剂"[不加权:扩展] OR "抗肿瘤药, 免疫"[不加权:扩展] OR “免疫治疗” [全部字段:智能] OR “免疫治疗靶点” [全部字段:智能] OR “免疫治疗方法” [全部字段:智能] OR “免疫治疗药物” [全部字段:智能] OR “免疫治疗方案” [全部字段:智能] OR “免疫治疗剂” [全部字段:智能] OR “免疫检查点抑制剂” [全部字段:智能] OR “抗肿瘤药,单克隆抗体” [全部字段:智能] OR “抗肿瘤药,免疫” [全部字段:智能] OR “单抗” [全部字段:智能] | 54705 |
| #3 | ((#1) AND (#2)) AND ( 动物[特征词]) | 8 |
| **CNKI** |  | **44 results** |
| #1 | (主题: 免疫治疗 + 免疫治疗靶点 + 免疫治疗方法 + 免疫治疗药物 + 免疫治疗方案 + 免疫治疗剂+ 免疫检查点抑制剂 + 抗肿瘤药单克隆抗体 + 抗肿瘤药,免疫+单抗) OR (篇关摘: 免疫治疗 + 免疫治疗靶点 + 免疫治疗方法 + 免疫治疗药物 + 免疫治疗方案 +免疫治疗剂+ 免疫检查点抑制剂 + 抗肿瘤药,单克隆抗体 + 抗肿瘤药,免疫+单抗(模糊)) | 38912 |
| #2 | (主题: 聚焦超声 + 聚焦超声消融治疗 + 聚焦超声热疗 + 强度聚焦超声+ 高强聚焦超声 + 聚焦超声消融术 + 高频聚焦超声 + 高强聚焦超声消融 +低强度聚焦超声(lifu'+ 合成孔径聚焦超声 +相控聚焦超声 + 高强度体外聚焦) OR (篇关摘: 聚焦超声 + 聚焦超声消融治疗 + 聚焦超声热疗 + 低强度聚焦超声+ 高强聚焦超声聚焦超声消融术 + 高频聚焦超声 + 高强聚焦超声消融 +低强度i聚焦超声(lifu)'+ 合成孔径聚焦超声 + 相控聚超声 + 高强度体外聚焦(模糊)) | 8138 |
| #3 | #1 AND #2 | 44 |
| **万方医学网(wangfang)** |  | **45 results** |
| #1 | (主题=(免疫治疗 OR 免疫治疗靶点 OR 免疫治疗方法 OR 免疫治疗药物 OR 免疫治疗方案 OR 免疫治疗剂OR免疫检查点抑制剂 OR 抗肿瘤药,单克隆抗体 OR 抗肿瘤药,免疫OR单抗)) OR 题名或关键词=(免疫治疗 OR 免疫治疗靶点 OR 免疫治疗方法 OR 免疫治疗药物 OR 免疫治疗方案 OR 免疫治疗剂OR免疫检查点抑制剂 OR 抗肿瘤药,单克隆抗体 OR 抗肿瘤药,免疫OR单抗) 资源类型：(中文期刊 OR 外文期刊) | 30460 |
| #2 | (主题=(聚焦超声 OR 聚焦超声消融治疗 OR 聚焦超声热疗 OR 低强度聚焦超声OR 高强聚焦超声 OR 聚焦超声消融术 OR 高频聚焦超声 OR 高强聚焦超声消融 OR 低强度聚焦超声 OR 合成孔径聚焦超声 OR 相控聚焦超声 OR 高强度体外聚焦超声 OR 高功率聚焦超声 OR 聚焦超声治疗 OR 聚焦超声消融 OR 聚焦超声刀 OR 聚焦超声波 OR 聚焦超声技术 OR 高强聚焦超声消融 OR 聚焦超声手术 OR 聚焦超声热疗 OR 体外高强度聚焦超声疗法 OR 体外高强度聚焦超声治疗 OR 高强度聚焦超声疗法 OR 高强度聚焦超声治疗)) OR 题名或关键词=(聚焦超声 OR 聚焦超声消融治疗 OR 聚焦超声热疗 OR 低强度聚焦超声OR 高强聚焦超声 OR 聚焦超声消融术 OR 高频聚焦超声 OR 高强聚焦超声消融 OR 低强度聚焦超声 OR 合成孔径聚焦超声 OR 相控聚焦超声 OR 高强度体外聚焦超声 OR 高功率聚焦超声 OR 聚焦超声治疗 OR 聚焦超声消融 OR 聚焦超声刀 OR 聚焦超声波 OR 聚焦超声技术 OR 高强聚焦超声消融 OR 聚焦超声手术 OR 聚焦超声热疗 OR 体外高强度聚焦超声疗法 OR 体外高强度聚焦超声治疗 OR 高强度聚焦超声疗法 OR 高强度聚焦超声治疗) 资源类型：(中文期刊 OR 外文期刊) | 9464 |
| #3 | #1 AND #2 | 45 |

## Supplementary Tables

a
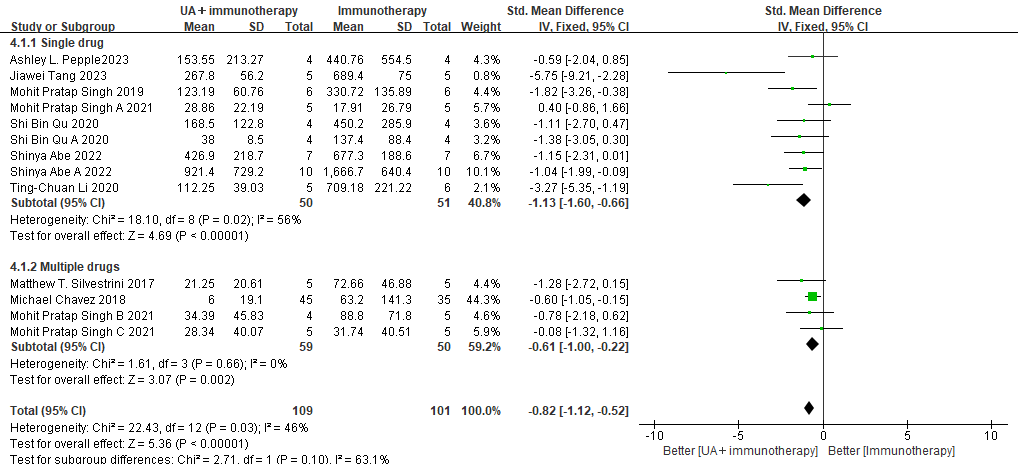


b
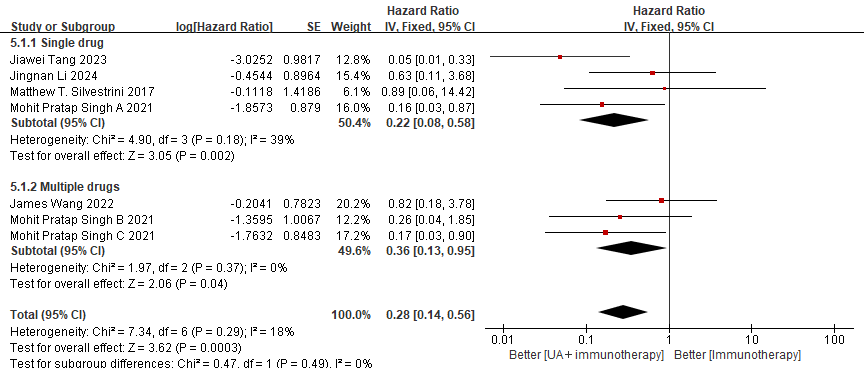


c
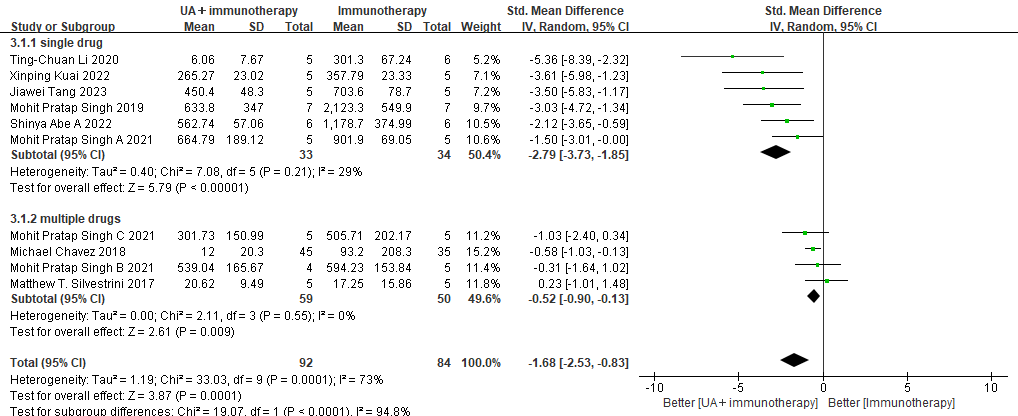


**Supplementary Figure 1.** Subgroup analysis of immune therapy outcomes is visualized using forest plots. The left side of the plot shows that the combination of focused ultrasound (UA) and immunotherapy provides a greater benefit, while the right-side highlights improved outcomes for the group receiving immunotherapy alone. a, the subgroup analysis of distant tumor volume reveals that the pooled standard mean difference (SMD) for both the multi-drug and single-drug subgroups are located on the left side of the forest plot, with no values crossing zero. b, the subgroup analysis of survival time reveals that the pooled hazard ratio (HR) for both the multi-drug and single-drug subgroups are located on the left side of the forest plot, with no values crossing one. c, the subgroup analysis of treated tumor volume reveals that the pooled standard mean difference (SMD) for both the multi-drug and single-drug subgroups are located on the left side of the forest plot, with no values crossing zero. The ◆ symbol represents the pooled effect size.

a
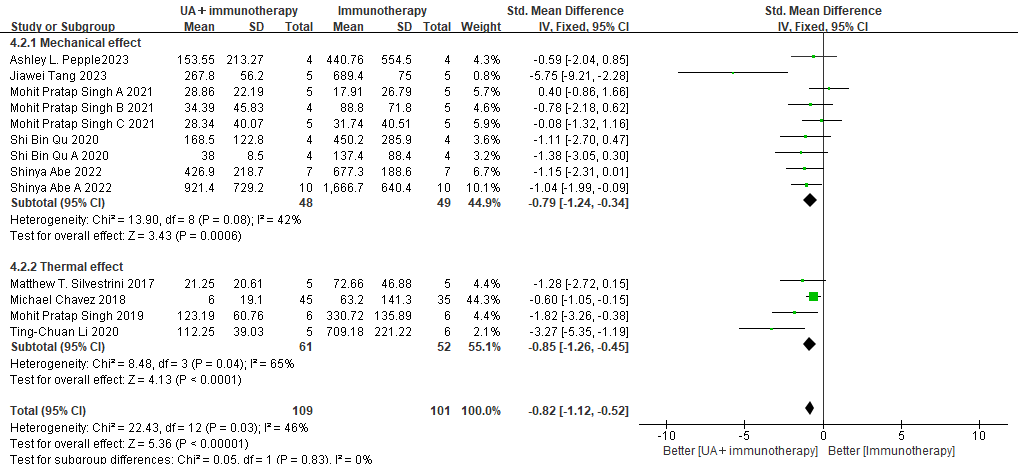


b
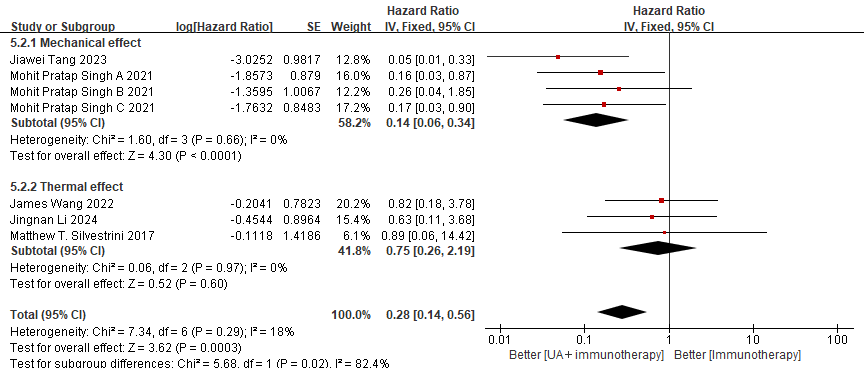


c
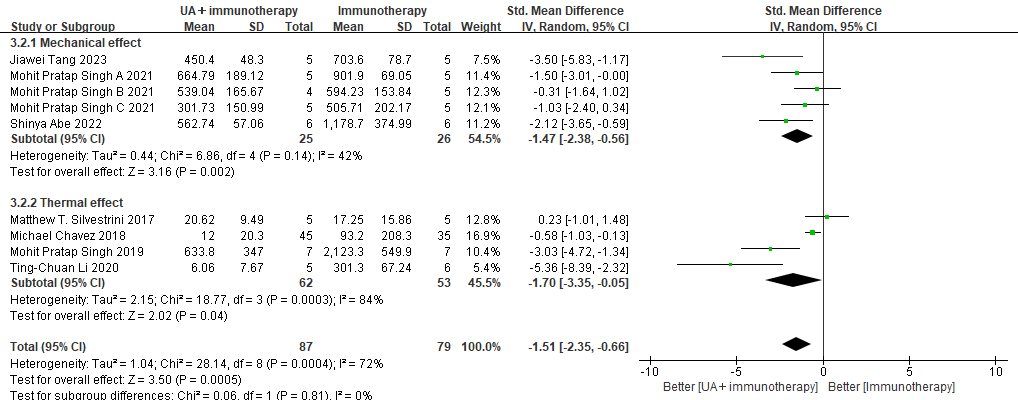


**Supplementary Figure 2.** Subgroup analysis of focused ultrasound effects outcomes is visualized using forest plots. The left side of the plot shows that the combination of focused ultrasound (UA) and immunotherapy provides a greater benefit, while the right-side highlights improved outcomes for the group receiving immunotherapy alone. a, the subgroup analysis of distant tumor volume reveals that the pooled standard mean difference (SMD) for both the mechanical effect and thermal effect subgroups are located on the left side of the forest plot, with no values crossing zero. b, the subgroup analysis of survival time reveals that the pooled hazard ratio (HR) for the mechanical effect subgroup falls entirely on the left side of the forest plot, with no values exceeding one. In contrast, the thermal effect group also appears on the left side of the plot, but its values cross one. c, the subgroup analysis of treated tumor volume reveals that the pooled standard mean difference (SMD) for both the mechanical effect and thermal effect subgroups are located on the left side of the forest plot, with no values crossing zero. The ◆ symbol represents the pooled effect size.

a
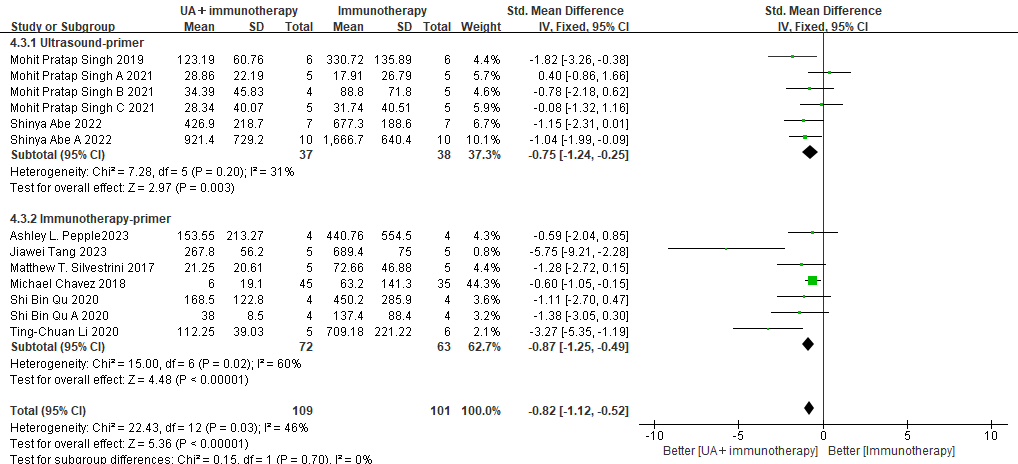


b
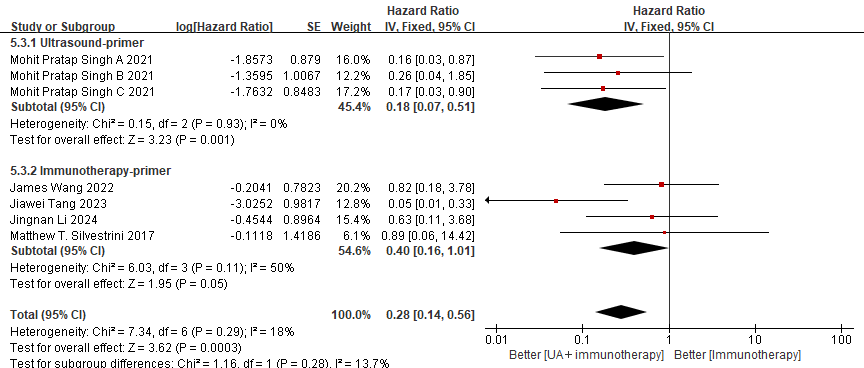


c
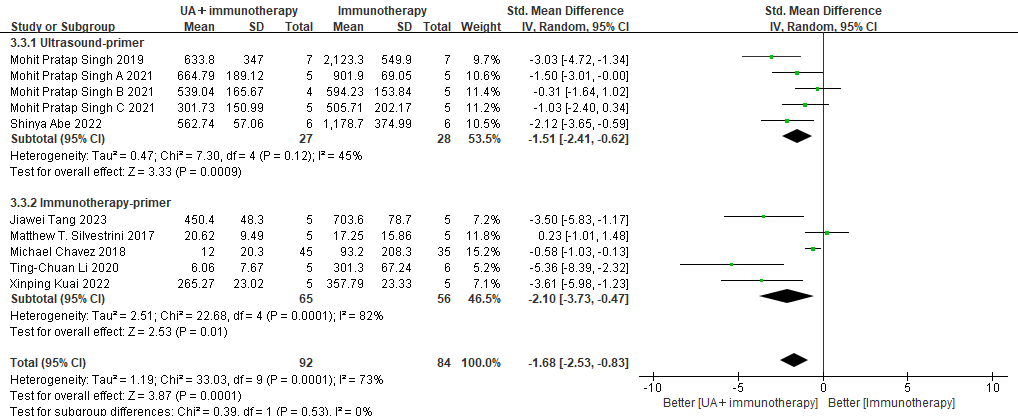


**Supplementary Figure 3.** Subgroup analysis of combination therapy sequence outcomes is visualized using forest plots. The left side of the plot shows that the combination of focused ultrasound (UA) and immunotherapy provides a greater benefit, while the right-side highlights improved outcomes for the group receiving immunotherapy alone. a, the subgroup analysis of distant tumor volume reveals that the pooled standard mean difference (SMD) for both the ultrasound-primer and immunotherapy-primer subgroups are located on the left side of the forest plot, with no values crossing zero. b, the subgroup analysis of survival time reveals that the pooled hazard ratio (HR) for the ultrasound-primer subgroup falls entirely on the left side of the forest plot, with no values exceeding one. In contrast, the immunotherapy-primer group also appears on the left side of the plot, but its values cross one. c, the subgroup analysis of treated tumor volume reveals that the pooled standard mean difference (SMD) for both the ultrasound-primer and immunotherapy-primer subgroups are located on the left side of the forest plot, with no values crossing zero. The ◆ symbol represents the pooled effect size.

a
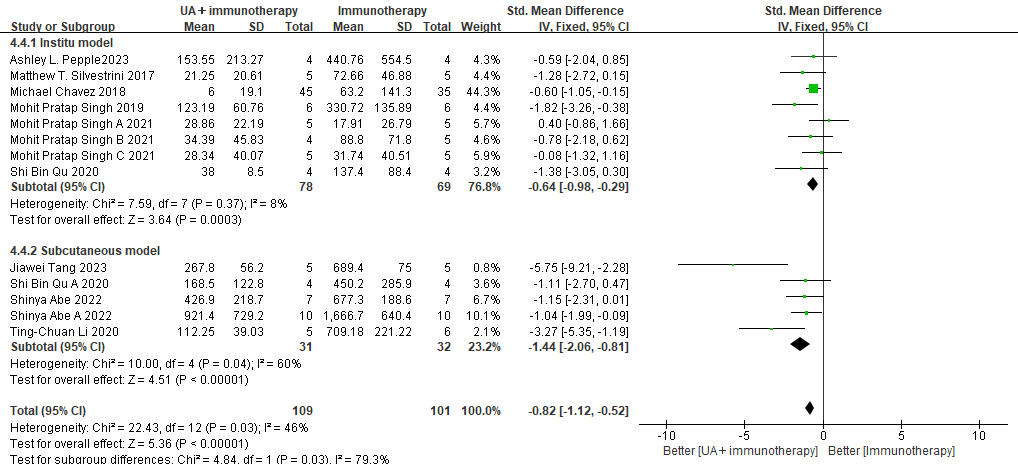


b
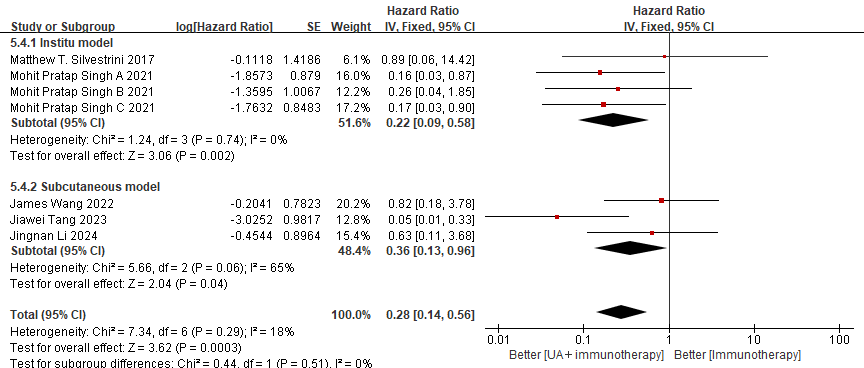


c
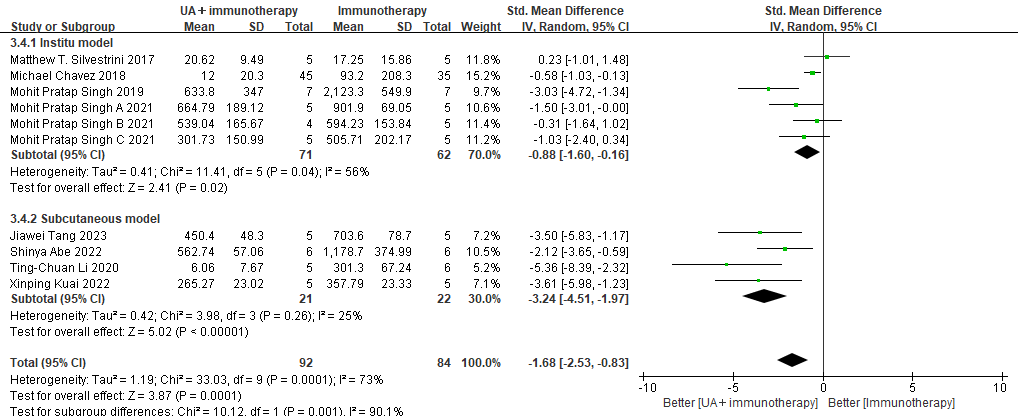
 **Supplementary Figure 4.** Subgroup analysis of tumor site outcomes is visualized using forest plots. The left side of the plot shows that the combination of focused ultrasound (UA) and immunotherapy provides a greater benefit, while the right-side highlights improved outcomes for the group receiving immunotherapy alone. a, the subgroup analysis of distant tumor volume reveals that the pooled standard mean difference (SMD) for both the in-situ model and subcutaneous model subgroups are located on the left side of the forest plot, with no values crossing zero. b, the subgroup analysis of survival time reveals that the pooled hazard ratio (HR) for both the in-situ model and subcutaneous model subgroups are located on the left side of the forest plot, with no values crossing one. c, the subgroup analysis of treated tumor volume reveals that the pooled standard mean difference (SMD) for both the in-situ model and subcutaneous model subgroups are located on the left side of the forest plot, with no values crossing zero. The ◆ symbol represents the pooled effect size.

a
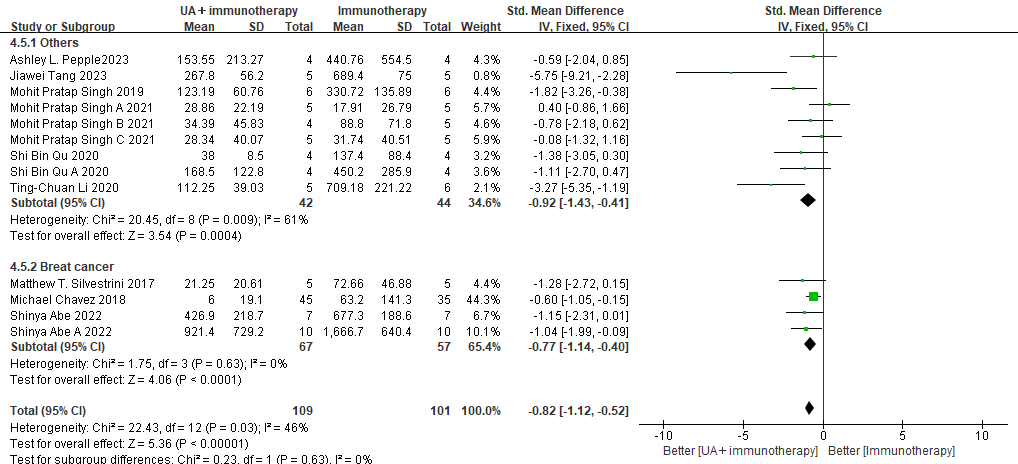


b


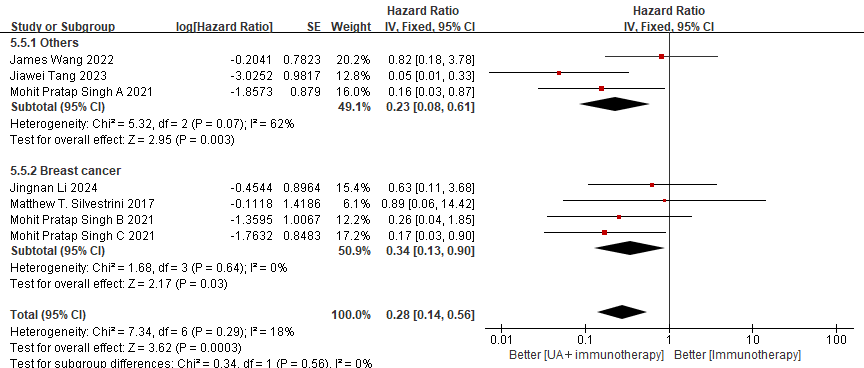


c
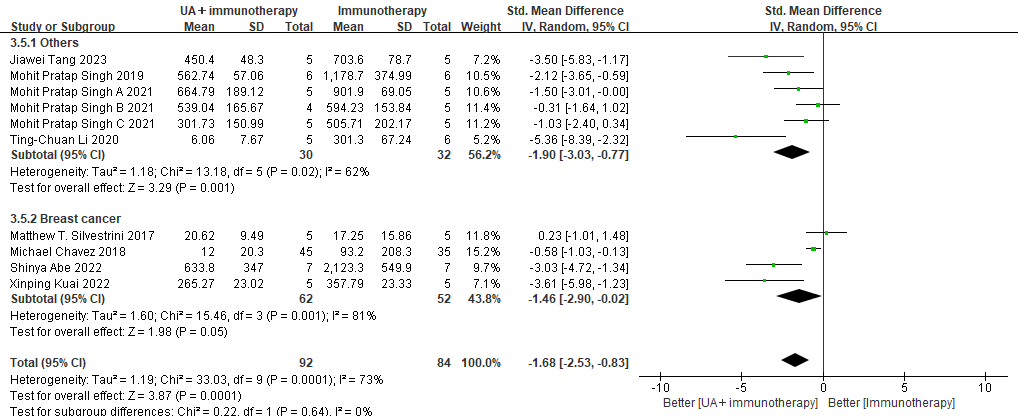


**Supplementary Figure 5.** Subgroup analysis of tumor type outcomes is visualized using forest plots. The left side of the plot shows that the combination of focused ultrasound (UA) and immunotherapy provides a greater benefit, while the right-side highlights improved outcomes for the group receiving immunotherapy alone. a, the subgroup analysis of distant tumor volume reveals that the pooled standard mean difference (SMD) for both the breast cancer and others subgroups are located on the left side of the forest plot, with no values crossing zero. b, the subgroup analysis of survival time reveals that the pooled hazard ratio (HR) for both the breast cancer and others subgroups are located on the left side of the forest plot, with no values crossing one. c, the subgroup analysis of treated tumor volume reveals that the pooled standard mean difference (SMD) for both the breast cancer and others subgroups are located on the left side of the forest plot, with no values crossing zero. The ◆ symbol represents the pooled effect size.

a

b

c

**Supplementary Figure6.** Sensitivity analysis. a, distant tumor volume; b, survival time; c, treated tumor volume.
